# Functional analyses of cotton (*Gossypium hirsutum* L.) immature fiber (*im*) mutant infer that fiber cell wall development is associated with stress responses

**DOI:** 10.1186/1471-2164-14-889

**Published:** 2013-12-17

**Authors:** Hee Jin Kim, Yuhong Tang, Hong S Moon, Christopher D Delhom, David D Fang

**Affiliations:** 1 Cotton Fiber Bioscience Research Unit, USDA-ARS-SRRC, 1100 Robert E. Lee Blvd., New Orleans, LA 70124, USA; 2 The Samuel Roberts Noble Foundation, Genomics Core Facility, Ardmore, OK 73401, USA; 3 Cotton Structure and Quality Research Unit, USDA-ARS-SRRC, 1100 Robert E. Lee Blvd., New Orleans, LA 70124, USA

**Keywords:** Abiotic and biotic stress, Cellular respiration, Cell wall development, Cotton, Ethylene, Reactive oxygen species, Transcriptome profile

## Abstract

**Background:**

Cotton fiber maturity is an important factor for determining the commercial value of cotton. How fiber cell wall development affects fiber maturity is not well understood. A comparison of fiber cross-sections showed that an immature fiber (*im*) mutant had lower fiber maturity than its near isogenic wild type, Texas marker-1 (TM-1). The availability of the *im* mutant and TM-1 provides a unique way to determine molecular mechanisms regulating cotton fiber maturity.

**Results:**

Transcriptome analysis showed that the differentially expressed genes (DEGs) in the *im* mutant fibers grown under normal stress conditions were similar to those in wild type cotton fibers grown under severe stress conditions. The majority of these DEGs in the *im* mutant were related to stress responses and cellular respiration. Stress is known to reduce the activity of a classical respiration pathway responsible for energy production and reactive oxygen species (ROS) accumulation. Both energy productions and ROS levels in the *im* mutant fibers are expected to be reduced if the *im* mutant is associated with stress responses. In accord with the prediction, the transcriptome profiles of the *im* mutant showed the same alteration of transcriptional regulation that happened in energy deprived plants in which expressions of genes associated with cell growth processes were reduced whereas expressions of genes associated with recycling and transporting processes were elevated. We confirmed that ROS production in developing fibers from the *im* mutant was lower than that from the wild type. The lower production of ROS in the *im* mutant fibers might result from the elevated levels of alternative respiration induced by stress.

**Conclusion:**

The low degree of fiber cell wall thickness of the *im* mutant fibers is associated with deregulation of the genes involved in stress responses and cellular respiration. The reduction of ROS levels and up-regulation of the genes involved in alternative respirations suggest that energy deprivation may occur in the *im* mutant fibers.

## Background

Cotton (*Gossypium* sp.) is the world’s most important natural fiber. Fiber quality is classified based on its physical properties such as length, strength, fineness, and maturity [1,2]. Among these properties, the fiber fineness and maturity are not well defined or understood [1,2]. The term of fiber fineness has been used to define fiber perimeter, diameter, cross-sectional area, linear density (mass per unit length), and specific fiber surface. Among them, the linear density is most often used to define fiber fineness by the textile industry. In plant physiological terms, fiber maturity refers to the degree of fiber cell wall thickness [2,3]. Since the fiber maturity and fineness determine the number of cotton fibers in a yarn, they directly affect yarn strength, performance, and dyeing efficiency [2,4]. Cotton fibers with either low or high maturity are classified as low grade for making yarns because less mature fibers with thin cell walls tend to be weak and easily broken during the spinning process, while overly mature fibers with thick cell walls produce coarse and thick yarns that are unfavorable to consumers. To measure fiber maturity and fineness, cell wall area (A) and perimeter (P) of multiple fibers need to be measured using the microscopic images from fiber cross-sections [2,3]. Absolute value of fiber maturity defined as circularity (θ) representing the degree of fiber cell wall development is calculated using the equation, θ = 4πA/P^2^[2-4]. Despite its superiority for measuring fiber maturity and fineness, microscopic image analysis has not been frequently used due to its long and laborious process. For a quick and automated assessment, fiber fineness and maturity have been indirectly measured as “micronaire” (MIC) that is determined by measuring air-flow resistance through a plug of cotton fibers of a given weight. Despite the MIC value representing a combination of fiber maturity and fineness of cotton fibers, the MIC is an effective way of measuring fiber maturity of commercial cotton varieties. Thus, textile industry and agricultural marketing services have used the MIC values as a key quality assessment parameter of determining the fiber maturity [1,2]. Changes in temperature, water content of soil, and mineral nutrition significantly affect the MIC values of cotton fibers: therefore, environmental factors affect MIC value of cotton fibers [1-5].

**Figure 1:**
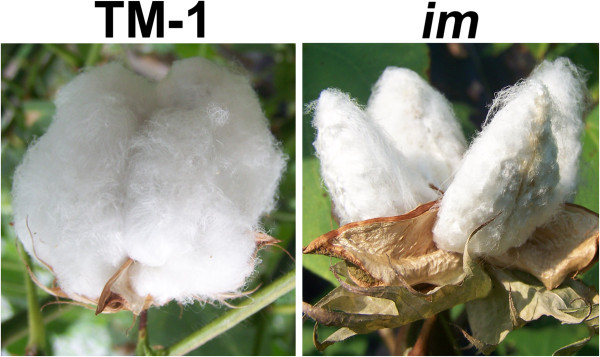
**Comparison of phenotypes from two near isogenic lines of *Gossypium hirsutum*.** Wild type Texas Marker-1 (TM-1) shows fluffy bolls, whereas immature fiber (*im*) mutant shows non-fluffy bolls.

When grown under normal environmental conditions, a wild type Upland cotton cultivar produces fluffy cotton bolls with MIC values ranging from 3.0 to 5.5 (Figure 1). When grown under severe stress conditions such as drought, cold temperature, or pathogens, the same cultivar produces non-fluffy bolls with MIC values less than 3.0 [6-8]. In the early 1970s, an immature fiber (*im*) mutant that produces non-fluffy bolls with low MIC fibers was discovered (Figure 1) [9]. The phenotype of the *im* mutant grown under normal field conditions resembles that of the wild type plant grown under severe stress [7,9-12]. This similarity suggested that the *im* mutation might be caused by a reduced ability of withstanding stress. Previously, we showed that the *im* gene mutation reduced fiber cell wall thickness, and the *im* gene located on chromosome 3 [11].

In the current paper we utilize a comparative genomics approach to analyze the *im* mutant and its near isogenic line (NIL) wild type TM-1 in order to better understand how the *im* gene affects fiber cell wall development. Our transcriptome results show that the *im* gene reduces the degree of fiber cell wall thickness by altering the expressions of genes involved in stress responses and cellular respiration.

## Results

### Comparison of *im* mutant and TM-1 wild type fibers

**Figure 2:**
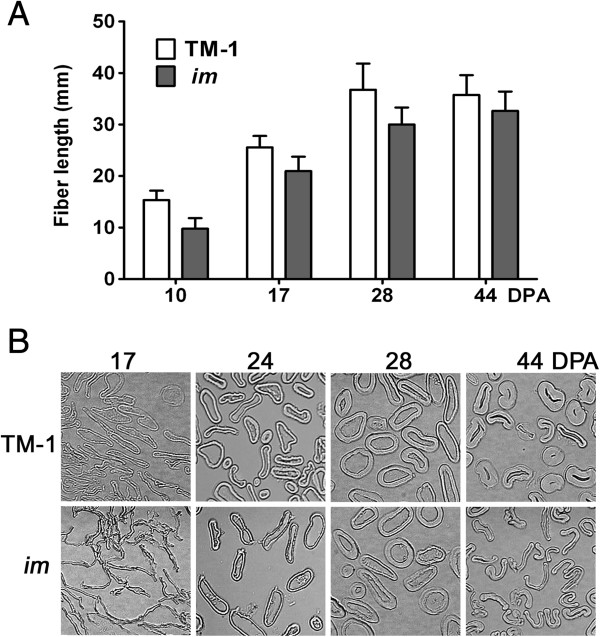
**Comparisons of fiber properties from TM-1 wild type and *im *mutant. A**. Fiber length comparison. The average fiber lengths of developing fibers at 10, 17, and 28 DPA were manually measured, and the average fiber length of mature fibers at 44 DPA was measured by AFIS. Average fiber lengths of developing fibers at each time point were calculated from two replicate samples with 30 ovules per replicate. The error bars represent SD. **B**. Microscopic image analyses. Cotton fibers at 17, 24, and 44 DPA from the TM-1 and *im* were cross-sectioned, embedded, and photographed.

In the cotton field where the two NILs were grown side by side under the same environmental conditions, wild type TM-1 showed a fluffy fiber phenotype, whereas *im* mutant had a non-fluffy boll (Figure 1). We manually measured fiber length and maturity from the developing fibers at four different developmental time points that represented the active elongation stage (10 days post anthesis, DPA), the transition stage (17 DPA) from elongation to secondary cell wall (SCW) biosynthesis stage, the active SCW biosynthesis stage (28 DPA), and the maturation stage (44 DPA) (Figure 2). Average fiber lengths from developing fibers (10, 17, and 28 DPA) of the *im* mutant were shorter (p value < 0.0001) than the equivalent fibers fromTM-1. TM-1 fibers elongated actively at 10 DPA (15.3 mm) and 17 DPA (25.6 mm), and reached maximal length at 28 DPA (36.8 mm). At the same DPAs, the average fiber lengths of the *im* mutant were 9.8 mm, 21.0 mm, and 30.0 mm that were 35.9%, 18.0% and 18.5% shorter than those of TM-1, respectively (Figure 2A). The final length of developed fibers from the *im* mutant was similar to that from TM-1 (Figure 2A).

Fiber cell wall thickness between TM-1 and *im* mutant were compared using a microscopic image analysis (Figure 2B). At 17 DPA, cross-sections of TM-1 fibers were circular with thin but detectable secondary cell walls, while *im* mutant fibers appeared linear due to a lack or very low level of SCW cellulose. At 24 DPA, fiber cross-sections of both NILs were circular but TM-1 fibers were thicker than the *im* fibers. At 44 DPA, the fiber cell wall in the *im* fibers was clearly thinner than that in the TM-1 (Figure 2B). Table 1 shows the results for the quantitative comparison of the degrees of fiber cell wall thickness (θ) between two NILs. These results clearly show that the *im* mutation greatly affects thickening of the secondary cell wall in both developing and mature fibers.

**Table 1:** **Relative ratio of circularities representing the degree of fiber cell wall development between TM-1 and *im *** **mutant**

**Fiber development**	***im***		**TM-1**		**Ratio (*im*/TM-1)**
	**Circularity**	**SD**	**Circularity**	**SD**	
17 DPA developing fibers	N.D.	N.D.	0.31	0.12	N.D.
24 DPA developing fibers	0.30	0.10	0.53	0.10	0.57
44 DPA mature fibers	0.41	0.15	0.61	0.15	0.67

### Comparative gene expression analysis of developing fibers from *im* mutant and TM-1 wild type

**Figure 3:**
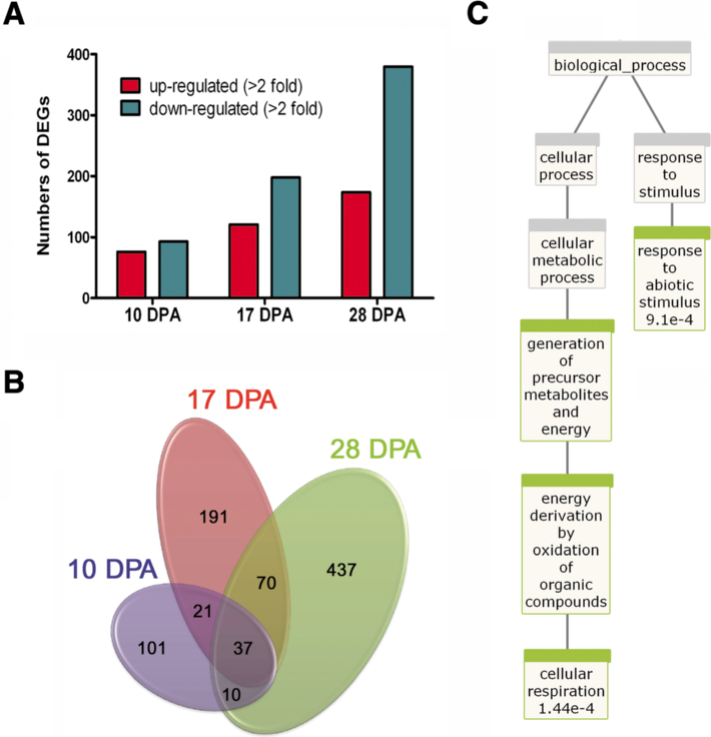
**Summary of microarray analysis comparing TM-1 wild type and *im *mutant. A**. Comparison of up- or down-regulated DEGs at 10, 17, and 28 DPA in the *im* mutant fibers. **B**. Venn diagrams representing the common DEGs among the three developmental stages in the *im* mutant. **C**. BioMaps enrichment analysis from the common DEGs at 10, 17, and 28 DPA in the *im* mutant fibers. Genes involved in abiotic stimulus response (p = 0.009) and cellular respiration (p = 0.000144) were enriched in the *im* mutant fibers.

Affymetrix cotton GeneChip genome arrays were used to identify differentially expressed genes (DEGs) in developing fibers from TM-1 and *im* mutant. Expression levels of transcripts were compared at the 10, 17, and 28 DPA. Among the 21,854 transcripts contained in an array chip, 867 unique transcripts were differentially expressed genes (DEGs) at all three time points from the *im* mutant fibers with more than a 2 fold difference in transcript abundance compared to TM-1 fibers (Additional file 1). At all three stages, the number of down-regulated transcripts in the *im* mutant was more than that of up-regulated transcripts (Figure 3A). Most of the DEGs were developmentally-regulated only at one of the specific cotton fiber developmental stages, whereas 37 DEGs were identified at all three stages (Figure 3B). Among the 37 DEGs, 19 DEGs have a high degree of sequence similarity to known *Arabidopsis* orthologs; these were annotated and further analyzed (Table 2). An enrichment analysis using BioMaps [13] showed that two biological processes involved in abiotic stimulus response (p = 0.009) and cellular respiration (p = 0.000144) were significantly enriched among the commonly identified DEGs (Figure 3C). Of the 19 DEGs, 10 DEGs are predicted to be involved in responses to abiotic or biotic stresses in *Arabidopsis*, 4 DEGs are in metal ion binding or transport, and 3 DEGs are in cellular respiration (Table 2).

**Table 2:** **Commonly identified DEGs at three different developmental stages of the *im *** **mutant fibers**

**Probe ID**	**Ratio (*im*/TM-1)**			**TAIR ID**	**Annotation**	**Biological process**
	**10 DPA**	**17 DPA**	**28 DPA**			
Ghi.3408.1.A1_at	5.65	55.57	148.77	At3g22370	Alternative oxidase 1	Abiotic response & respiration
Ghi.659.1.S1_at	21.64	18.08	18.16	At1g74920	Aldehyde dehydrogenase	Abiotic response (salt & water)
GhiAffx.24025.1.S1_at	18.07	27.24	17.44	At4g12560	F-box domain protein	Biotic stress response
Ghi.576.3.A1_x_at	7.97	31.71	14.64	At5g40690	EF-Hand 1	Calcium-binding
Ghi.9252.1.A1_at	5.00	2.65	2.45	At3g24590	Signal peptidase 1	Biotic stress response (virus)
GhiAffx.32033.1.A1_s_at	4.78	2.99	4.28	At1g55790	Unknown function (DUF2431)	Metal ion transport
GhiAffx.3843.1.A1_at	4.53	4.05	4.81	At3g11600	Response to karrikin	Abiotic stress
Ghi.8619.1.A1_at	4.38	2.68	2.43	At4g27220	NB-ARC disease resistance	Biotic stress response
Ghi.9264.2.A1_s_at	3.20	4.88	4.38	At5g64210	Alternative oxidase 2	Respiration
Ghi.3288.2.S1_at	3.19	5.05	4.45	At5g07990	Cytochrome P450 protein	Abiotic stress response
Ghi.3591.1.A1_s_at	3.19	3.98	2.84	At1g65820	Glutathione s-transferase	Transport
Ghi.8369.3.A1_at	2.15	2.16	2.48	At2g35510	SRO1, similar to RCD one 1	Abiotic stress response
Gra.778.1.A1_s_at	0.43	0.35	0.48	At4g32250	Protein kinase	Senescence
Ghi.4198.4.S1_s_at	0.43	0.37	0.36	At2g40010	Ribosomal protein L10	Protein synthesis
GhiAffx.22316.1.S1_at	0.39	0.28	0.46	At5g55160	Small ubiquitin-like modifier 2	Abiotic stress response
GhiAffx.59002.1.S1_at	0.36	0.07	0.24	At1g19530	Involve in anaerobic respiration	Respiration
GhiAffx.43892.1.A1_at	0.30	0.39	0.22	At5g39790	5′-AMP-activated protein kinase	Carbohydrate metabolism
Ghi.7402.1.S1_at	0.14	0.29	0.39	At5g25460	Unknown function (DUF642)	Abiotic stress response
GhiAffx.2217.1.S1_at	0.06	0.14	0.11	At2g43360	BIO2, BIOB, Radical SAM protein	Zinc binding & meristem growth

### Annotation of differentially expressed genes

The expression of genes involved in ethylene biosynthesis and pathway was changed substantially in the *im* mutant (Table 3). The six transcripts encoding *1-aminocyclopropane-1-carboxylate (ACC) oxidase* (Ghi.798.1.S1_s_at, Gra.2141.1.S1_s_at, Ghi.6953.1.S1_s_at, Ghi.6502.1.S1_at, Ghi.8023.1.S1_at, and Ghi.7921.1.S1_x_at), a key enzyme producing ethylene hormone and a large number of *AP2-ERF transcription factors* were down-regulated in the developing fibers of the *im* mutant. In contrast, *gibberellin 20 oxidase* (Ghi.6164.1.A1_at), a key enzyme involved in gibberellins biosynthesis, was highly up-regulated in the *im* mutant fibers (17 and 28 DPA). The expression of transcripts involved in cell wall biosynthesis including *xyloglucan endotransglycosylase* (XET, GraAffx.8958.1.S1_s_at, Ghi.6236.1.S1_s_at, GhiAffx.63628.1.S1_at, and Ghi.4532.1.A1_at), *pectin* (GhiAffx.25797.1.S1_s_at and Ghi.5186.1.A1_at) and *sucrose synthase* (Ghi.8667.1.A1_at) were reduced in the *im* mutant fibers. In addition to *AP2-ERFs*, a large number of *WRKY, NAC* and *MYB* transcription factors involved in stress response and fiber development were also down-regulated in the *im* mutant (Table 3). Numerous genes known to be involved in the response to abiotic and biotic stress were differentially regulated. Moreover, genes involved in protein synthesis and lipid metabolism known to be regulated by stress were also differentially regulated (Table 3).

**Table 3:** **Annotation of DEGS in developing fibers of the *im *** **mutant**

**Ratio (*im*/TM-1)**				
**Probeset ID**	**10 DPA**	**17 DPA**	**28 DPA**	**Annotation**
**A. Ethylene & GA biosynthesis**				
Ghi.798.1.S1_s_at	2.04	0.54	0.19	1-aminocyclopropane-1-carboxylate (ACC) oxidase
Ghi.6164.1.A1_at	1.02	380.56	122.59	Gibberellin 20 oxidase
**B. Cell wall metabolism**				
GhiAffx.63628.1.S1_at	0.71	0.48	0.24	Xyloglucan endotransglycosylase
GhiAffx.25797.1.S1_s_at	1.58	0.39	0.38	Pectinesterase family protein
Ghi.5186.1.A1_at	1.37	0.03	0.09	Pectin methylesterase
Ghi.8667.1.A1_at	1.14	0.28	0.26	Sucrose synthase
Gra.569.1.A1_s_at	0.69	1.25	2.01	β-amylase
**C. Stress related genes**				
**C-1. Stress related transcription factors**				
Ghi.3815.1.A1_at	1.58	0.50	0.33	WRKY transcription factor
GhiAffx.30199.1.S1_at	1.27	0.87	0.28	WRKY transcription factor
Ghi.10747.1.S1_at	1.57	0.11	0.40	Ethylene-responsive element binding protein ERF5
GhiAffx.6226.1.S1_at	0.87	0.50	0.45	AP2-ERF transcription factor
GbaAffx.196.1.A1_s_at	0.90	0.16	0.32	AP2-ERF transcription factor
Ghi.7874.1.S1_s_at	0.94	0.15	0.28	AP2-ERF transcription factor
Ghi.6538.1.S1_at	1.05	0.50	0.31	NAC transcription factor
Ghi.3264.2.A1_s_at	1.03	0.26	0.51	NAC transcription factor
Ghi.4770.1.S1_at	0.90	1.28	0.25	myb family transcription factor
GraAffx.1750.1.S1_s_at	0.89	1.35	0.25	myb family transcription factor
Ghi.8087.1.S1_s_at	0.99	1.50	0.21	myb-like transcription factor 3
**C-2. Temperature stress related genes**				
GhiAffx.41577.1.S1_s_at	0.19	0.52	3.73	17.3 kDa class I heat shock protein
GhiAffx.25696.1.S1_at	0.12	0.30	2.92	22.0 kDa class IV heat shock protein precursor
Ghi.4957.1.S1_s_at	1.48	2.66	2.58	70 kda heat shock protein
GhiAffx.25696.1.S1_at	0.12	0.30	2.92	22.0 kDa class IV heat shock protein precursor
GhiAffx.2754.1.A1_at	1.00	0.80	6.79	Cytosolic class small heat shock protein type 2
GhiAffx.34159.1.A1_a_at	0.24	0.79	6.21	Heat shock protein 18
GhiAffx.41577.1.S1_s_at	0.19	0.52	3.73	Low molecular weight heat shock protein
Ghi.8364.1.A1_at	0.77	0.24	0.31	Heat shock protein
**C-3. Water stress related genes**				
Ghi.4632.1.A1_at	1.08	1.32	0.42	Early-responsive to dehydration expressed
Gra.840.1.A1_at	0.95	0.92	0.21	Alcohol dehydrogenase a
GarAffx.29310.2.S1_s_at	0.90	0.93	0.19	Alcohol dehydrogenase a
Ghi.10646.1.S1_s_at	0.76	0.93	0.31	Osmotin precursor
Ghi.1478.1.S1_s_at	0.65	0.48	0.44	Aquaporin protein PIP1
GraAffx.33059.2.S1_s_at	0.58	0.48	0.44	Aquaporin protein PIP1
GhiAffx.52815.1.S1_at	0.90	5.59	4.86	Putative aquaporin PIP1
Ghi.766.1.S1_at	0.91	5.21	3.87	Aquaporin PIP1
**C-4. Pathogen responsive genes**				
GhiAffx.41878.1.S1_at	39.35	11.57	1.27	TIR-NBS-LRR type disease resistance protein
Ghi.8619.1.A1_at	4.38	2.68	2.43	TIR-NBS-LRR type disease resistance protein
Ghi.8798.1.S1_at	1.05	1.11	0.37	TIR-NBS-LRR type disease resistance protein
Ghi.1498.1.S1_s_at	0.80	0.78	0.41	Receptor protein kinase clavata1
Ghi.5675.1.A1_x_at	0.48	0.47	0.30	14 kda proline-rich protein
GbaAffx.198.1.S1_at	1.69	0.87	0.27	Proline-rich protein
GbaAffx.198.1.S1_s_at	1.60	0.85	0.26	Proline-rich protein
Ghi.7872.1.S1_s_at	0.59	1.22	0.07	Proline-rich glycoprotein
Ghi.6523.1.S1_s_at	1.37	0.41	0.36	Pathogenesis-related protein 10
GbaAffx.201.1.S1_s_at	1.49	0.35	0.36	Pathogenesis-related protein 10
**D. Ribosomal proteins**				
GraAffx.15288.1.S1_s_at	0.86	0.45	0.78	Ribosomal protein L3A
Ghi.4198.4.S1_s_at	0.43	0.37	0.36	60S acidic ribosomal protein P0
**E. Lipid metabolism**				
Ghi.6324.1.A1_x_at	1.01	1.28	0.24	Lipid transfer protein
Gra.378.1.A1_s_at	0.29	0.82	0.18	Lipid transfer protein
Ghi.6175.2.S1_s_at	0.29	0.83	0.16	Lipid transfer protein
Ghi.576.3.A1_x_at	7.97	31.71	14.65	Glycerol-3-phosphate acyltransferase
Ghi.8520.1.S1_at	1.13	2.04	2.44	Delta-6 desaturase

### Validation of differentially expressed genes

**Figure 4:**
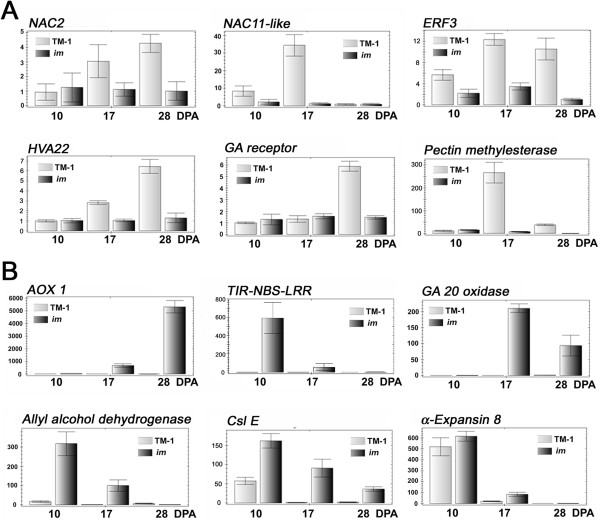
**Validation of array data by RT-qPCR analysis. A**. Down-regulated genes in the *im* mutant. *NAC2 transcription factor* (Ghi.3264.2.A1_s_at), *NAC 11-like transcription factor* (Ghi.6896.1.A1_s_at), *ethylene responsive element binding factor 3 (ERF3) transcription factor* (Ghi.10747.1.S1_at), *HVA22 protein* (GhiAffx.8267.1.A1_s_at), *GA receptor* (Gra.1544.1.A1_s_at) and *pectin methylesterase* (Ghi.5186.1.A1_at). **B**. Up-regulated genes in the *im* mutant. *alternative oxidase 1* (*AOX 1*, Ghi.3408.1.A1_at), *TIR-NBS-LRR resistance protein* (GhiAffx.41878.1.S1_at), *GA 20 oxidase* (Ghi.6164.1.A1_at), *allyl alcohol dehydrogenase* (Ghi.8523.1.A1_at), *cellulose synthase-like protein* (*CslE*, Ghi.3562.1.A1_at), and *α-expansin 8* (Ghi.2039.1.S1_x_at). All RT-qPCR analyses were performed with three biological replications at each time point with five technical replications. The error bars represent SD.

The expression patterns of the DEGs identified from the *im* mutant fibers were validated with quantitative real time PCR analysis (RT-qPCR). Differential expressions of 26 DEGs showed in the microarray analysis were validated by RT-qPCR. As shown in Figure 4A, genes involved in stress response were down-regulated in the *im* mutant fibers. These include the following: the *GhNAC2* transcription factor (Ghi.3264.2.A1_s_at, ACI15342) previously shown to be differentially regulated in cotton leaves by cold stress and abscisic acid [14], an unknown *NAC* transcription factor (Ghi.6896.1.A1_s_at) showing 76% sequence similarity to *GhNAC11* (AGC97441), an *ethylene responsive element binding factor* (*EREBP3* or *ERF3*) that is a transcription repressor (Ghi.10747.1.S1_at) binding to GCC box or pathogenesis-related promoter element [15], a *HVA22* protein (GhiAffx.8267.1.A1_s_at) known to be induced by abscisic acid and stress in barley aleurone cells [16], a *gibberellic acid (GA) receptor* (Gra.1544.1.A1_s_at) also known as *GA insensitive dwarf1* (*GID1*) that restricts plant growth [17], and a *pectin methylesterase* (Ghi.5186.1.A1_at) that is an important cell wall enzyme involved in plant tolerance to chilling/freezing by the brassinosteroid hormone pathway [18]. Other stress response genes were up-regulated in developing fibers of the *im* mutant (Figure 4B). These include the following genes: *alternative oxidase 1* (AOX 1, Ghi.3408.1.A1_at) known as a target and regulator of stress responses [19], *Toll/Interleukin-1 receptor nucleotide-binding sites Leu-rich repeat* (*TIR-NBS-LRR*, GhiAffx.41878.1.S1_at) involved in biotic and abiotic stress response [20], a *GA 20 oxidase* (Ghi.6164.1.A1_at) induced by abiotic stress [21], *allyl alcohol dehydrogenase* (Ghi.8523.1.A1_at), *cellulose synthase-like protein* (*CslE*, Ghi.3562.1.A1_at), and *α-expansin 8* (Ghi.2039.1.S1_x_at).

### Gene ontology analyses of differentially expressed genes

To identify the potential biological processes governing differential expressions of genes in the *im* mutant fibers, we analyzed the identified 867 DEGs using each of three different Gene Ontology (GO) term enrichment tools: Singular Enrichment Analysis (SEA), Parametric Analysis of Gene set Enrichment (PAGE), and MapMan [22,23]. The GO categories identified by the three analyses (p-value ≤ 0.05) were separated into four different classes (Table 4).

**Table 4:** Three gene ontology term enrichment analyses with differentially expressed genes

**Class**	**SEA**	**MapMan**	**PAGE**
**Class 1: Stress responding process**	• GO:0071466, cellular response to xenobiotic stimulus (10 DPA; 6 DEGs)	Bin 20, stress (10 DPA; 25 DEGs)	GO:0006979, response to oxidative stress (10 DPA; 14 DEGs)
		Bin 20.2, abiotic stress (10 DPA; 21 DEGs)	GO:0050896, response to stimulus (28 DPA; 103 DEGs)
**Class 2: Cellular respiration process**	• GO:0006120, GO:0042775, mitochondrial electron transport/ATP synthesis (28 DPA; 6 DEGs)	Bin 9, mitochondrial electron transport/ATP synthesis (28 DPA; 7 DEGs)	GO:0045333, GO:0015980, GO:0031966, cellular respiration/energy derivation/mitochondrial membrane (28 DPA; 13 DEGs)
**Class 3: Cell wall related process**	GO:0005985, sucrose metabolic process (28 DPA; 9 DEGs)		GO:0008194, GO:0016758, GO:0016757, glycosyltransferase activity (28 DPA; 10 DEGs)
**Class 4: Transport and ion binding**	GO:0006814, sodium ion transport (28 DPA; 6 DEGs)		GO:0008270, zinc ion binding (10 DPA; 14 DEGs)
	GO:0015992, proton transport (28 DPA; 16 DEGs)		GO:0005506, iron ion binding (17 & 28 DPA; 37 DEGs)
	GO:0006771, riboflavin metabolic process (10 DPA; 5 DEGs)		GO:0046914, transition metal ion binding (17 & 28 DPA; 56 DEGs)

Class 1, “stress responding processes”, were commonly identified at 10 DPA by all three methods. In addition, the PAGE analysis identified 103 DEGs at 28 DPA fibers as genes responding to environmental stimuli.

Class 2, “cellular respiration processes”, were identified by all three methods at 28 DPA. All identified DEGs in this class are involved in mitochondrial electron transport and ATP synthesis through a classical cytochrome C oxidase (COX) respiratory pathway that generates ATP and ROS [24]. The class 1 and 2 identified by SEA, MapMan, and PAGE were consistent with the results by the BioMaps analysis with the 37 common DEGs that were overlapped in 10, 17, and 28 DPA fibers (Tables 2 and 4).

Class 3, “cell wall related processes” were identified by SEA and PAGE analyses at 28 DPA, but not by MapMan analysis (Table 4). SEA analysis identified 9 DEGs involved in sucrose metabolic pathways, and PAGE analysis identified 10 DEGs involved in glycosyltranserase activity catalyzing a transfer of a glycosyl or hexosyl group from a UDP-sugar (Additional file 2).

Class 4, “transporting processes” were identified by SEA and PAGE analyses. SEA analysis identified sodium ion transport (GO:0006814) and proton transport (GO:0015992), whereas PAGE analysis identified genes involved in zinc and iron binding (GO:0008270 and GO:0005506), and other transition metal ion binding (GO:0046914) (Additional file 2).

### Class 1: Stress responding processes

**Figure 5:**
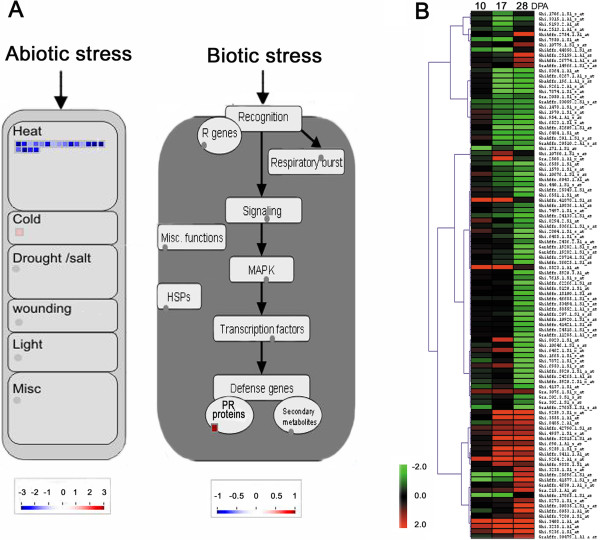
**Class 1 involved in stress response in developing fibers of the *im *mutant. A**. MapMan-based visualization of the DEGs involved in abiotic and biotic stress in the *im* mutant fibers at 10 DPA. Blue and red color squares represent down- and up-regulated genes with a *log*2 scale, respectively. **B**. Hierarchical clustering of 103 DEGs identified as stimulus responsive genes by PAGE analysis in the *im* mutant fibers at 28 DPA. The detailed annotation data and the magnified heatmap are described at the Additional file 3.

The stress responding DEGs identified by MapMan (Table 4) in 10 DPA fibers were further analyzed by Scavenger module and ImageAnnotator [23]. These analyses showed that 20 DEGs were abiotic stress related genes, whereas one DEG was a biotic stress related gene (Figure 5A). Of the 20 abiotic stress related DEGs identified, 19 DEGs were predicted to be regulated by heat and 1 DEG by cold (Figure 5A). The one biotic stress related DEG was a PR-protein in the downstream portion of the biotic stress pathway, but no other biotic stress related gene was identified in the upstream of the pathway.

Figure 5B showed a hierarchical clustering of the 103 DEGs identified as genes responding to environmental stimuli from 28 DPA fibers by the PAGE analysis. Among them, dozens of heat shock proteins, transcripts responding to drought and osmotic adjustment (Ghi.4632.1.A1_at, Ghi.1478.1.S1_s_at, GraAffx.33059.2.S1_s_at, GhiAffx.52815.1.S1_at, and Ghi.766.1.S1_at), and flood (Gra.840.1.A1_at and GarAffx.29310.2.S1_s_at) were differentially regulated. Several alternative oxidases (Ghi.3408.1.A1_at and Ghi.9264.2.A1_s_at) were also identified in the list (Figure 5B and Additional file 3).

### Class 2: Cellular respiration process in mitochondria of *im* mutant

**Figure 6:**
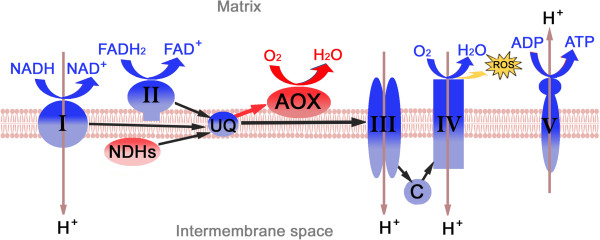
**Class 2 involved in cellular respiration and election transport chain in plant mitochondria.** Genes involved in a classical cytochorme c oxidase (COX) respiration pathway generating energy and ROS were down-regulated (blue), whereas genes involved in alternative oxidase respiration pathways were up-regulated (red) in the *im* mutant fibers. AOX, alternative oxidase; I, NADH:dehydrogenase or NADH-plastoquinone oxidoreductase; II, succinate:ubiquione oxidoreductase; III, cytochorme bc_1_ complex; IV, cytochrome c oxidase; V, ATP synthase; NDHs, rotenone-insensitive NAD (P) H dehydrogenase; ROS, reactive oxygen species; UQ, ubiquione pool.

Microarray analysis identified 12 DEGs involved in a mitochondrial classical cytochrome *c* oxidase (COX) respiration pathway producing energy and reactive oxygen species (ROS) (Table 5). As shown in Figure 6, plant mitochondria possess additional pathways that are mediated by alternative oxidase (AOX) and the alternative NAD (P) H dehydrogenases (NDHs). They enable respiration in the presence of cyanide and rotenone [25]. Four DEGs involved in AOX pathway and one DEG involved in alternative NDH were identified from the microarray analysis (Table 5). Most genes involved in the COX pathway were down-regulated 3–5 fold at 17 DPA and a few of them were up-regulated 2–4 fold at 28 DPA (Table 5). In contrast, all four AOX from alternative pathway were greatly up-regulated during the SCW thickening stages (17 and 28 DPA). Among them, one AOX (AOX1, Ghi.3408.1.A1_at) that was similar to *Arabidopsis* stress responding AOX1 was up-regulated 149 fold by the microarray (Table 5) and 5,000 fold by the RT-qPCR assay (Figure 4) at 28 DPA in the *im* fibers. The rotenone insensitive NADH dehydrogenase involved in another alternative respiratory pathway was also up-regulated during the *im* mutant fiber development (Table 5).

**Table 5:** **Differentially expressed class 2 genes involved in cellular respiration at developing fibers of** ***im*** **mutant**

		**Affy. Probes**	**Annotation**	**Transcript fold (*im*/TM-1)**		
**No.**	**Pathways**			**10 DPA**	**17 DPA**	**28 DPA**
1	COX Complex I	GhiAffx.53261.1.A1_at	NADH dehydrogenase subunit 4	1.0	0.8	2.3
2	COX Complex I	GhiAffx.4260.1.S1_at	NADH-plastoquinone oxidoreductase subunit 6	1.1	0.4	2.4
3	COX Complex I	GhiAffx.9732.1.A1_at	NADH-plastoquinone oxidoreductase	1.1	0.4	4.6
4	COX Complex I	GhiAffx.61810.1.S1_at	NADH dehydrogenase	1.2	0.3	1.4
5	COX Complex I	GhiAffx.18012.1.S1_at	NADH dehydrogenase 6	0.3	0.2	0.9
6	COX Complex I	GhiAffx.45916.1.A1_s_at	NADH dehydrogenase subunit 2	1.3	0.5	3.5
7	COX Complex III	GhiAffx.38970.1.S1_at	Cytochrome b/b6	1.0	0.3	1.8
8	COX Complex V	GhiAffx.819.1.S1_at	ATPase, F0 complex, subunit A protein	1.1	0.4	2.3
9	COX Complex V	GraAffx.25748.1.A1_s_at	ATPase, F0 complex, subunit A protein	1.3	0.3	2.0
10	COX Complex V	GhiAffx.44858.1.S1_at	ATPase, F1 complex, alpha subunit protein	0.4	0.2	1.2
11	COX Complex V	GhiAffx.52777.1.S1_at	ATP synthase epsilon chain	1.0	0.3	0.9
12	COX Complex V	GhiAffx.7848.1.S1_at	ATP synthase subunit beta	1.2	0.3	1.9
13	Alternative AOX	Ghi.3408.1.A1_at	Mitochondrial alternative oxidase 1	5.7	55.6	148.8
14	Alternative AOX	Ghi.9264.3.S1_s_at	Mitochondrial alternative oxidase 2	2.5	5.9	5.3
15	Alternative AOX	Ghi.9264.2.A1_s_at	Mitochondrial alternative oxidase 2	3.2	4.9	4.4
16	Alternative AOX	GraAffx.30195.1.A1_s_at	Mitochondrial alternative oxidase 2	1.5	2.2	2.2
17	Alternative NDH	Ghi.3524.2.A1_at	Rotenone-insensitive NADH dehydrogenase	2.7	3.9	5.3

### Comparison of differentially regulated genes controlling production of reactive oxygen species from *im* mutant and TM-1 fibers

Under stress conditions, AOX1 was suggested to prevent excess ROS formation by bypassing electron transfer from the COX respiratory pathway [19,26-28] (Figure 6). Based on the results showing high levels of AOX1 in the developing fibers of the *im* mutant, we predicted reduced activity of the COX pathway that generated energy and ROS.

**Figure 7:**
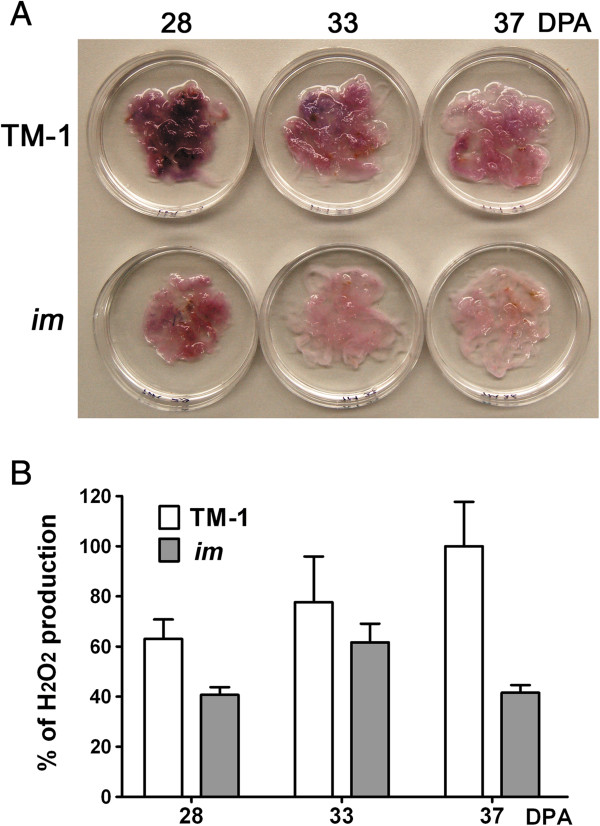
**Comparison of ROS levels of developing fibers from TM-1 and *im *mutant. A**. Superoxide radicals were detected by NBT staining from freshly harvested developing fibers at 28, 33, and 37 DPA from TM-1 and *im* mutant. **B**. Relative productions of H_2_O_2_ in developing cotton fibers were quantitatively measured from freshly harvested developing fibers at 28, 33, and 37 DPA from TM-1 and *im* mutant. Three biological replications and three technical readings at each time point were performed. The error bars represent SD.

To test if the up-regulation of AOX1 affects the ROS production in developing fibers of the *im* mutant, we measured the levels of superoxide radicals from developing fibers between TM-1 and the *im* mutant. Superoxide radicals were detected by semi-quantitatively staining freshly harvested developing fibers (28, 33, and 37 DPA) with NBT [29]. The intensity of the purple color was used as a measure of superoxide amounts present in the fibers [29]. Lower intensity of purple color in the developing *im* mutant fibers at 28, 33, and 37 DPA than the TM-1 fibers suggested a decreased amount of superoxide radicals in the *im* mutant fibers (Figure 7A). The levels of hydrogen peroxide were also quantitatively compared from developing fibers (28, 33, and 37 DPA) between TM-1 and the *im* mutant [30]. The content of hydrogen peroxide in developing fibers of TM-1 increased as fibers became mature from 28 to 33 and 37 DPA (Figure 7B). At these different stages of fiber development, the hydrogen peroxide content in the *im* mutant was consistently lower (35.4%, 20.6%, and 58.4%) than that in the TM-1 fibers (Figure 7B). Based on the results of the reduced ROS levels along with the elevated levels of AOX in the developing fibers of the *im* mutant, we concluded that the activity of the classical COX respiration pathway generating energy and ROS were reduced in developing fibers of the *im* mutant.

## Discussion

### Comprehensive GO enrichment analyses identified two common biological processes deregulated in the *im* mutant

Although there are no standard GO enrichment analyses established for the cotton transcriptome, GO enrichment analyses in other plants are useful and powerful tools for interpretation of the cotton transcriptome data [8,31,32]. However, such analyses may identify substantially different annotated genes [33]. To improve the GO analyses, we analyzed the transcriptome data using four different methods (BioMaps, SEA, MapMan, and PAGE). The biological processes and the GO categories (p-value ≤ 0.05) identified by each analysis were different to some extent, but all four methods identified two common biological processes, *i.e.*, stress responding process (class 1) and the cellular respiration process (class 2) that were deregulated in the *im* mutant (Tables 2 and 4).

### The *im* gene affects fiber elongation and cell wall development

The *im* mutant fibers elongated more slowly and had thinner cell walls than the TM-1 fibers (Figure 2). The thin cells of the *im* mutant fibers exhibited less efficient SCW biosynthesis. Forty five DEGs identified as being involved in reducing the degree of SCW development were down-regulated at the active SCW stage in the *im* mutant fibers (Additional file 4). Among the 45 DEGs identified, a gene encoding *sucrose synthase* is one of the main genes involved in SCW biosynthesis. Several transcriptional factors (*HVA22* and *ERFs*) involved in the stress-related phytohormones (abscisic acid and ethylene) pathways may be involved in reducing the degree of fiber cell wall development in the *im* mutant (Additional file 4).

We also identified 30 candidate genes involved in fiber elongation at 28 DPA in the *im* mutant fibers. They were up-regulated at 28 DPA in the *im* mutant fibers (Additional file 5). Among them, *α-expansin 8*, GA and brassinosteroid pathway related genes, and several heat shock proteins might help to continue the elongation of the *im* mutant fibers at 28 DPA (Figure 4B). The transcriptome profiles also showed that the DEGs involved in stress response (class 1) and cellular respiration (class 2) are commonly identified as being involved in regulating elongation (10 DPA), transition (17 DPA) and SCW biosynthesis stages (28 DPA). Therefore the deregulation of these genes in the *im* mutant fibers would be expected to contribute to the mutant phenotype (Tables 2 and 4). Based on these results, we concluded that the *im* gene affected both fiber elongation and SCW biosynthesis.

### The *im* gene most likely interferes with the ability of the *im* mutant to respond to stress

**Figure 8:**
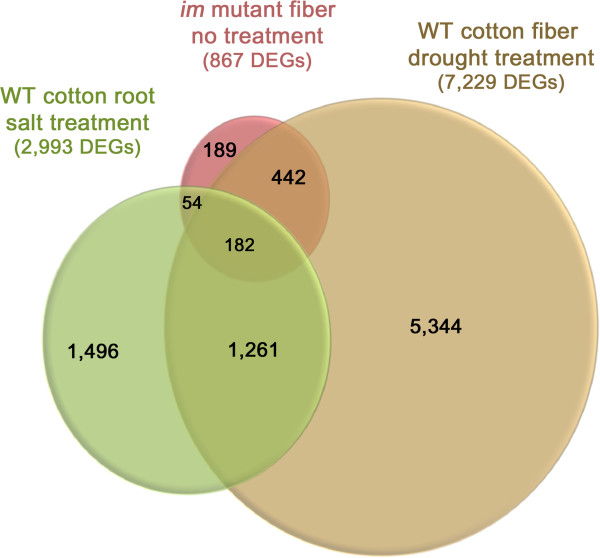
**Venn diagrams representing the common DEGs among *im *mutant fibers without additional stress treatment, wild type cotton roots with salt treatment **[32]** and wild type cotton fibers with drought treatment **[8]**.** The DEGs showing 2 fold difference (p value ≤0.05) were compared one another.

All four GO enrichment analysis methods commonly identified genes involved in the stress response process (class 1) as being deregulated in the *im* mutant. Furthermore, the transcriptome profiles of the *im* mutant showed significant enrichments of the same DEGs previously identified as being affected in wild type cotton exposed to drought or salt stress [8,32,34]. Padmalatha et al. [8] reported that 4,522 transcripts (20.7%) of the total 21,854 transcripts on the Affymetrix chip were regulated by three fold (p value ≤0.01)in the drought stressed wild type cotton fibers. Yao et al. [32] reported that 2,993 transcripts (12.5%) among the 23,977 cotton transcripts printed on the Affymetrix chip were regulated by two fold differences (p value ≤0.05 )in the salt stressed wild type cotton roots. When the microarray results from the *im* mutant fibers are compared with the results from the wild type cottons stressed by drought or salt using a 2 fold difference criterion (p value ≤ 0.05), we found that 624 DEGs (72%) and 236 DEGs (27%) of the 867 DEGs identified in the *im* mutant fibers overlapped the DEGs in the drought stressed cotton fibers and the salt stressed cotton roots, respectively (Figure 8). Figure 8 showed that 182 DEGs from the *im* mutant fibers were common to the drought stressed wild type cotton fibers and salt stressed wild type cotton roots. Among these common DEGs, four ACC oxidases for ethylene biosynthesis, two ethylene responsive transcription factors for ethylene signal pathway, and three AOXs for alternative respiration pathway for controlling ROS levels were included (Additional file 6). These results suggest that the DEGs identified from the *im* mutant fibers are involved in some aspect of the control of common stress response pathways [8,19,32,35-40].

### The *im* gene is associated with the reduction of reactive oxygen species

Further evidence for the *im* gene being involved in stress response regulation is the fact that the mutant had reduced amounts of superoxide radicals and hydrogen peroxide compared to those in wild type TM-1 fibers (Figure 7). ROS production mainly occurs during mitochondrial cellular respiration process. The lower ROS levels in the *im* mutant fibers correlate with the finding that an alternative oxidase (AOX) involved in the respiration was the most significantly up-regulated in the *im* mutant fiber (Figure 4B). AOX was originally identified from a study of thermogenic respiration in *Arum maculatum* and other plants where heat is produced, but no ATP is generated [41]. AOX was suggested to be an enzyme able to reduce the level of ROS that could damage living cells, but which are generated as byproducts during ATP production from the classical COX respiration pathway [24,25,42]. AOX is also known as a “stress-induced protein” since it was induced by both abiotic and biotic stress such as chilling, wounding, drought, osmotic and nutrient stress, and pathogen [19,25,35,36]. In cotton plants, AOX was also induced by abiotic stress [32]. In *Arabidopsis*, there are two classes of AOX: AOX1 and AOX2 [36]. The expression level of AOX1 was induced by stress, whereas expression patterns of AOX2 were tissue specific and developmentally regulated. All four cotton AOX genes contained in the microarray chips were up-regulated in developing fibers of the *im* mutant during fiber development (Table 5). Among them, a cotton AOX gene (Ghi.3408.1.A1_at) with 91% sequence similarity to *Arabidopsis* AOX1A (AT3G22370) and AOX1B (AT3G22360) was highly up-regulated in the *im* mutant, whereas the other three cotton AOX genes showed high similarity with *Arabidopsis* AOX2 and were moderately (2–5 fold) up-regulated (Table 5). Over-expression of AOX1 was previously found to decrease ROS production whereas a lack of AOX in tobacco resulted in high cellular levels of ROS [43]. Consistent with this suggested function of AOX, developing fibers of the *im* mutant which contained high levels of AOX had reduced levels of ROS (Figure 7).

### The *im* gene may cause energy deprivation

AOX is also suggested to regulate energy balance and metabolic fluctuations in response to abiotic stress [24,44]. Unlike the COX pathway generating ATP required for cell growth and maintenance, AOX only produces heat without generating ATP [24,25]. Thus, energy deprivation in plants is a consequence caused by abiotic stresses reducing the activity of the COX respiratory pathway and activating the AOX respiratory pathway in mitochondria. When energy deprivation occurs in plant cells, transcript levels of genes involved in protein synthesis, cell wall biosynthesis, sucrose metabolism, transporters, chromosome and histone modification, and phytohormone signal pathways are changed [45]. The transcriptome profile obtained from developing fibers of *im* mutant clearly showed the same alteration of transcriptional regulation that happened during energy deprivation (Table 3). Transcript levels of ribosomal proteins responsible for protein synthesis and XET, pectinesterase, pectin methylesterase, and sucrose synthase involved cell wall biosynthesis were all reduced. The class 4 including sodium/proton ion transport, and several metal ion binding identified from developing fibers of the *im* mutant fibers may facilitate recycling processes of the molecules that are degraded from starch and cell walls.

### The *im* gene might be involved in stress sensing, signaling, and responding pathways

The transcriptome profiles from the *im* mutant fibers and abiotic stressed wild type cotton plants [8,31,32] showed many biotic stress responding genes that were previously classified as defense mechanism related genes or pathogenesis related (PR) genes although they were not affected by treatment of cotton with pathogenic organisms (Table 3). Recent studies have shown that many biotic stress-responding genes were also involved in abiotic stress responses [20,46-50]. TIR-NBS-LRR, one of the resistant (R) proteins known to be involved in defense against pathogenic organisms was also reported to be sensitive to temperature changes in *Arabidopsis*[20]. *Arabidopsis* pathogenesis-related (PR) proteins known as molecular markers for biotic stress caused by pathogens were also involved in response to abiotic stress factors such as light and high concentration of salts [50]. Thus, we suspect that the pathogen responsive genes and PR proteins described in Table 3 and Figure 5A were likely to respond to abiotic stress. Therefore, we cannot rule out an important contribution to the results from abiotic stress and these might affect the expression levels of the DEGs identified in the *im* mutant fibers. Abiotic stress from drought, high salinity, and low temperature are well known factors affecting fiber maturity measured as MIC value [1,2,5,8].

Like the *im* mutant fibers in cotton, *Arabidopsis* SCW cellulose deficient mutants were reported to have deregulated expressions of stress related genes [51,52]. The stress related DEGs identified in the *im* mutant might be caused by impairments of SCW cellulose biosynthesis in the *im* mutant fibers as found in the *Arabidopsis* cellulose deficient mutants [51,52]. The *im* gene might be also involved in sensing or signaling rather than only in controlling the response to stress. Similar to a receptor-like kinase (THESEUS1) responsible for the sensing cell wall integrity and deregulating defense genes in *Arabidopsis* cellulose deficient mutants [53], unknown cell wall sensors might involve in sensing the thin cell wall and/or increasing sensitivity to stress in the *im* mutant fibers. The thin and impaired cell walls of the *im* mutant fibers might increase the stress effect. Phytohormone (abscisic acid and/or ethylene) signaling pathways related to stress responses might be also involved in reducing the degree of cell wall development in the *im* mutant fibers.

## Conclusion

**Figure 9:**
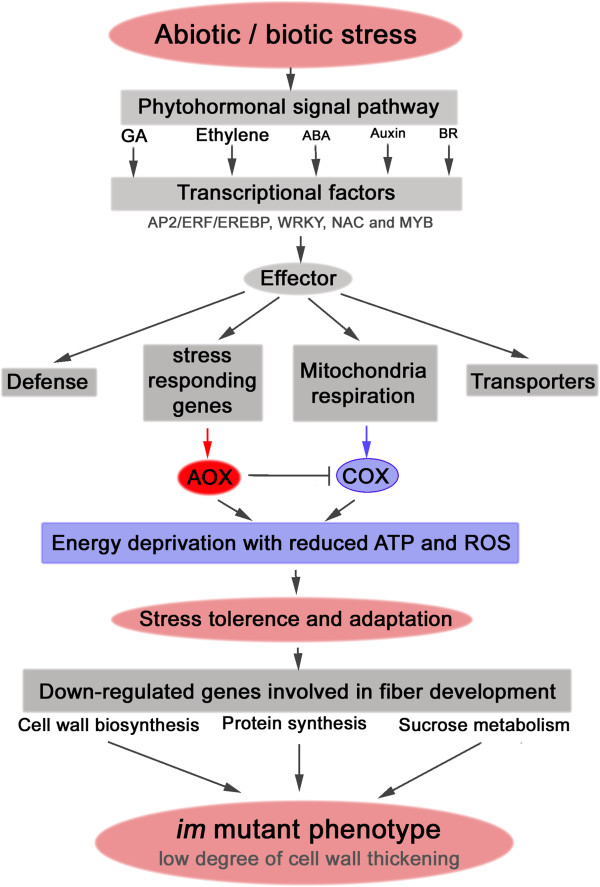
**Model for reducing the degree of fiber cell wall development in the *im *** **mutant.**

Based on the results from microarray comparison of the cotton *im* mutant to a near isogenic wild type, we identified differentially expressed genes in developing fibers of the *im* mutant that are consistent with the loss of secondary cell wall in the mutant (Figure 9). Our results suggest that genes controlling stress responses are affected in the mutant. Our data provide evidence that phytohormone signal pathways and their transcription factors controlling these pathways are deregulated in the mutant. Some of these genes concomitantly affect cellular respiration, production of defense proteins, and ion transporters. The alternative respiration pathway induced by stress reduced the levels of ROS that were originally generated by the classical COX respiration pathway in mitochondria. These up-regulated alternative respirations processes may provide stress adaptation to the *im* mutant and in turn cause energy deprivation. Expression of genes involved in cell wall biosynthesis, protein synthesis, and sucrose metabolism are reduced. As a result, the *im* mutant fibers are thin and immature. Our analyses suggest that the reduced ability to withstand stress in the *im* mutant may result in non-fluffy phenotype and the low degree of fiber cell wall thickness. Our results provide novel insights on genes involved in the interplay of stress and fiber maturity.

## Methods

### Plant material

Two cotton NILs, *G. hirsutum* Texas Marker-1 (TM-1) and immature mutant (*im*) were grown side by side in a field of USDA-ARS in New Orleans, LA for multiple years between 2006 and 2011. Cotton flowers were tagged at day of anthesis. Two biological replicates of cotton bolls from TM-1 and *im* mutant were harvested at 10, 17, 24, 28, and 44 DPA before 9 A.M. The developing fibers from 10–30 ovules that were harvested from 2–4 different cotton plants used for each biological replication. For RNA extraction, the immediately frozen fibers with liquid nitrogen were used. For image analysis, the intact ovules associated with developing fibers were stored in 50% ethanol. For measuring fiber properties, the developing fibers were manually ginned from the ovules and dried in 40°C incubator. For microarray transcriptome analyses, two biological replications at three different developmental time points between the two NILs grown in year 2011 were used. For verifications of the microarray results, the fiber samples harvested in multiple years (2006, 2008, and 2011) were used. During the all processes from planting, tagging, harvesting, and ginning, the two NILs grown side by side were equivalently treated at the same time. Standard conventional field practices were applied during growing season. The soil type in New Orleans was Aquent dredged over alluvium in an elevated location to provide adequate drainage.

### Fiber property measurements

Mature fibers from the two NILs were ginned using a laboratory roller gin, whereas developing fibers that were too fragile to be ginned by the roller gin were manually collected. Before the measurements of fiber properties, fibers were pre-equilibrated with 65% humidity and 21°C for 48 hours. The average fiber properties of five tests were determined by the Cotton Fiber Testing Lab in USDA-ARS-SRRC located in New Orleans, LA. Mean fiber length (Lw) from mature fibers was obtained from five replicates with 5000 fibers per replicate using Advanced Fiber Information System (USTER Technologies Inc., Knoxville, TN). Fiber lengths from developing fibers (10, 17, and 28 DPA) were manually measured [54]. The distance from the chalazal end of the ovule to the tip of the relaxed fibers by warm acidic water was measured to the nearest 0.01 mm with a digital caliper. Average fiber lengths of developing fibers at each time point were calculated from two replicate samples with 30 ovules per replicate. Statistical analyses and construction of graphs were performed using two-way ANOVA and Prism version 5 software (Graph-Pad Software, Inc., San Diego, CA).

### Image analysis to measure fiber maturity

Developing fibers (17, 24, and 28 DPA) were immediately immersed into 50% ethanol to maintain moisture for keeping original fiber structure. To prevent young and fragile fibers from being damaged by dryness, developing fibers were never dried until they were embedded. Matured fibers were harvested from cotton bolls when bolls were open at 44 DPA. Mature fibers were completely dried and embedded for microscopic image analysis. Both mature and developing fibers were thin-section cut, and photographed using the method previously described [11]. The cross-sections were analyzed using the image analysis software designed for measuring cross-sectional areas, perimeters and calculate circularity [55]. Average wall area (A) excluding lumen and perimeter (P) of the fiber cross sections were calculates from three hundred individual results for each fiber cross-section. Circularity (θ) representing the degree of fiber cell wall development was calculated using the equation, θ = 4πA/P^2^[3,4].

### RNA extraction from cotton fibers

Developing cotton fibers (10, 17, and 28 DPA) were manually ginned from cotton ovules and frozen with liquid nitrogen immediately. Total RNA was extracted from the frozen fibers using the Sigma Spectrum™ Plant Total RNA Kit (Sigma-Aldrich, St. Louis, MO) with DNase1 digestion according to the manufacturer’s protocol. The quality and quantity of total RNA were determined using a NanoDrop 2000 spectrophotometer (NanoDrop Technologies Inc., Wilmington, DE) and an Agilent Bioanalyzer 2100 (Agilent Technologies Inc., Santa Clara, CA).

### Microarray hybridizations and data analysis

The microarray experiments were conducted by following the minimum information about a microarray experiment (MIAME) guidelines [56]. Affymetrix GeneChip® Cotton Genome Arrays (Affymetrix Inc., Santa Clara, CA) representing 21,854 cotton transcripts were used to compare expression levels between TM-1 and *im* mutant. For each sample 500 ng of cotton fiber RNA was utilized for labeling using the Affymetrix GeneChip® 3′ IVT Express Kit and Cotton Genome Array hybridizations were performed according to standardized Affymetrix protocols. The developmental time-points from two NILs were 10, 17, and 28 DPA with two biological replicates at each time-point. Procedures for data normalization and assessment of statistically and biologically significant genes were performed as described previously [57]. The Affymetrix microarray dataset was deposited in the ArrayExpress database with the expression number E-MEXP-3901. Statistical analyses were performed to identify significantly differentially expressed genes in fibers of each cotton NIL at each time point with p value ≤ Bonferroni corrected P value of 0.05/N where N is numbers of probe set on the chip. Primary annotation was performed with the latest version of the Affymetrix annotation (Release 33), and additional annotations were performed using Blast2GO [58], and blastx in The *Arabidopsis* Information Resource (http://www.arabidopsis.org/Blast/) and *Gossypium raimondi*i genome sequence (http://www.phytozome.net/cotton.php). Gene Ontology (GO) enrichment and visualization of microarray results were performed according to the described methods [13,22,59]. For the SEA and PAGE analyses, the identified DEGs were compared with *Gossypium raimondii* genome [60]. For the BioMaps and MapMan analyses, *Arabidopsis* orthologues of the identified DEGs from the *im* mutant were compared with *Arabidopsis* genome sequences (http://www.arabidopsis.org/index.jsp). For statistical analyses and GO enrichment, the p-value cutoff for significance was 0.05.

### Reverse transcription quantitative real-time PCR (RT-qPCR)

The experimental procedures and data analysis related to RT-qPCR were performed according to the Minimum Information for Publication of Quantitative Real-Time PCR Experiments (MIQE) guidelines [61]. The cDNA synthesis reactions were performed using the iScript™ cDNA Synthesis Kit (Bio-Rad Laboratories, Hercules, CA) according to the manufacturer’s instructions with 1 μg of total RNA per reaction used as template. Control cDNA synthesis reactions to check for genomic DNA contamination during RT-qPCR consisted of the same template and components as the experimental reactions without the reverse transcriptase enzyme. Thirty seven pairs of specific primers were designed from twenty nine DEGs for validation of the microarray results. The RT-qPCR reactions were performed with iTaq™ SYBR® Green Supermix (Bio-Rad Laboratories) in a Bio-Rad CFX96 real time PCR detection system. Thermal cycler parameters for RT-qPCR were as follows: 95°C 3 minutes, 50 cycles of 95°C 15 seconds, 60°C 30 seconds. Dissociation curve was generated and used to validate that a single amplicon was present for each RT-qPCR reaction. The calculations for amplification efficiencies of the target and reference genes, and the relative quantifications of the different target gene transcript abundance were performed using the comparative Cq method as described in the ABI Guide to Performing Relative Quantitation of Gene Expression Using Real-Time Quantitative PCR (Applied Biosystems, Foster City, CA) with the following modification: the average of three reference gene Cq values was determined by taking the geometric mean which was used to calculate the ΔCq values for the individual target genes. The endogenous reference genes used in the RT-qPCR reactions were the *18S rRNA* (U42827), *ubiquitin-conjugating protein* (AI730710), and *α-tubulin 4* (AF106570). The reference and target gene primer sequences are shown in Additional file 7. Three biological replications and five technical replications were used for each time-point sample.

### Detection of superoxide radicals and hydrogen peroxide

Superoxide radicals were detected *in situ* by nitroblue tetrazolium (NBT) staining [29]. Freshly harvested developing fibers (28, 33, and 37 DPA) from TM-1 and *im* mutant plants were vacuum infiltrated for 5 min with 50 mM potassium phosphate buffer (pH 7.8) containing 0.1% NBT and 10 mM sodium azide and incubated at room temperature for 10, 30, and 60 min. The reactions were stopped by transferring the fibers into 95% ethanol. Relative productions of H_2_O_2_ in developing cotton fibers were quantitatively compared between TM-1 and *im* mutant using a xylenol orange assay [30,62]. Freshly harvested cotton fibers (28, 33, and 37 DPA) of the same weight were immediately vacuum infiltrated in a freshly prepared solution containing 25 mM FeSO_4_, 25 mM (NH_4_)_2_SO_4_, 2.5 M H_2_SO_4_, 100 mM sorbitol, and 125 μM xylenol orange and then incubated in room temperature for 2 h. Hydroperoxides were reduced by ferrous ions in acid solution and formed ferric product-xylenol orange complex that is detected spectrophotometrically at 560 nm.

## Abbreviations

AFIS: Advanced fiber information system; AOX: Alternative oxidase; COX: Cytochrome c oxidase; DEG: Differentially expressed gene; DPA: Days post anthesis; GO: Gene ontology; MIC: Micronaire; NBT: Nitroblue tetrazolium; NDHs: NAD (P) H dehydrogenases; NIL: Near-isogenic line; PAGE: Parametric analysis of gene set enrichment; ROS: Reactive oxygen species; RT-qPCR: Reverse transcription quantitative polymerase chain reaction; SCW: Secondary cell wall; SEA: Singular enrichment analysis.

## Competing interests

The authors declare that they have no competing interests.

## Authors’ contributions

HJK conceived the research, collected samples, determined ROS levels, analyzed fiber properties and microarray data, wrote and edited the manuscript. DDF conceived the research and edited the manuscript. YT performed statistical analysis and analyzed the microarray data. HSM extracted total RNAs and verified microarray results with qPCR analyses. CDD was responsible for microscopic image analysis. All authors read and approved the final manuscript.

## Supplementary Material

Additional file 1 **Annotation of differentially expressed genes.** Annotation and ratio of expression levels of differential expressed genes in the developing fibers between *im* mutant and TM-1 wild type at 10, 17, and 28 DPA. Click here for file

Additional file 2 **Classes 3 and 4.** Probeset ID identified as class 3 (sucrose metabolic process and gylcosyltransferase activity) and class 4 (transports and metal ion binding processes) by GO term enrichment analyses. Click here for file

Additional file 3 **Detailed annotation of the stimulus responding 103 DEGs s (Figure** 5**B) identified by PAGE analysis in the *im *** **mutant at 28 DPA.** GO enrichment analysis of the stimulus responding 103 DEGs in the *im* mutant at 28 DPA and a magnified heatmap described at the Figure 5B. Click here for file

Additional file 4 **45 candidate genes involved in reducing the degree of SCW development at 28 DPA of the *im *** **mutant fibers.** Common DEGs between the down-regulated DEGs in the *im* mutant fibers at 28 DPA compared with the TM-1 fibers at 28 DPA (*im* 28 DPA vs. TM-1 28 DPA) and up-regulated DEGs in the TM-1 fibers at 28 DPA compared with TM-1 at 10 DPA (TM-1 28 DPA vs. TM-1 10 DPA). Click here for file

Additional file 5 **30 candidate genes involved in fiber elongation at 28 DPA of the *im *** **mutant fibers.** Common DEGs between the up-regulated DEGs in the *im* mutant fibers at 28 DPA compared with the TM-1 fibers at 28 DPA (*im* 28 DPA vs. TM-1 28 DPA) and up-regulated DEGs in the TM-1 fibers at 10 DPA compared with TM-1 at 28 DPA (TM-1 10 DPA vs. TM-1 28 DPA). Click here for file

Additional file 6 **Common DEGs between *im *** **mutant fibers without additional stress treatment and wild type cotton tissues with salt or drought treatment.** Probeset ID commonly identified from *im* mutant fibers without additional stress treatment, wild type cotton roots with salt treatment, and wild type cotton fibers with drought treatment. Click here for file

Additional file 7 **qPCR primer sequences.** Forward and reverse primer sequences for quantitative PCR analysis. Click here for file

## References

[B1] Bradow JM Davidonis GH Quantitation of fiber quality and the cotton production-processing interface: A physiologist’s perspective J Cotton Sci 2000 14 34 64

[B2] Wakelyn PJ Bertoniere NR French AD Thibodeaux DP Triplett BA Rousselle M-A Goynes WR Jr Edwards JV Hunter L McAlister DD Cotton fiber chemistry and technology, vol. 17 2010 New York: CRC Press

[B3] Thibodeaux DP Evans JP Cotton fiber maturity by image analysis Text Res J 1986 14 2 130 139 10.1177/004051758605600211

[B4] Thibodeaux DP Rajasekaran K Development of new reference standards for cotton fiber maturity J Cotton Sci 1999 14 188 193

[B5] Haigler CH Zhang D Wilkerson CG Biotechnological improvement of cotton fibre maturity Physiol Plant 2005 14 3 285 294 10.1111/j.1399-3054.2005.00480.x

[B6] Marsh PB Guthrie LR Butler ML The influence of weathering and of microorganisms on the aqueous-extract pH of cotton fiber Text Res J 1951 14 8 565 579 10.1177/004051755102100804

[B7] Kohel RJ McMichael SC Immature fiber mutant of upland cotton Crop Sci 1990 14 2 419 421 10.2135/cropsci1990.0011183X003000020038x

[B8] Padmalatha KV Dhandapani G Kanakachari M Kumar S Dass A Patil DP Rajamani V Kumar K Pathak R Rawat B Genome-wide transcriptomic analysis of cotton under drought stress reveal significant down-regulation of genes and pathways involved in fibre elongation and up-regulation of defense responsive genes Plant Mol Biol 2011 14 3 223 246 22143977 10.1007/s11103-011-9857-y

[B9] Kohel RJ Quisenberry JE Benedict CR Fiber elongation and dry weight changes in mutant lines of cotton Crop Sci 1974 14 3 471 474 10.2135/cropsci1974.0011183X001400030040x

[B10] Kohel R Stelly D Yu J Tests of six cotton (Gossypium hirsutum L.) mutants for association with aneuploids J Hered 2002 14 2 130 132 10.1093/jhered/93.2.130 12140273

[B11] Kim HJ Moon HS Delhom CD Zeng L Fang DD Molecular markers associated with the immature fiber (im) gene affecting the degree of fiber cell wall thickening in cotton (Gossypium hirsutum L.) Theor Appl Genet 2013 14 1 23 31 10.1007/s00122-012-1956-x 22890806

[B12] Wang C Zhang T Guo W The im mutant gene negatively affects many aspects of fiber quality traits and lint percentage in cotton Crop Sci 2013 14 1 27 37

[B13] Katari MS Nowicki SD Aceituno FF Nero D Kelfer J Thompson LP Cabello JM Davidson RS Goldberg AP Shasha DE VirtualPlant: a software platform to support systems biology research Plant Physiol 2009 14 2 500 515 20007449 10.1104/pp.109.147025 PMC2815851

[B14] Meng C Cai C Zhang T Guo W Characterization of six novel NAC genes and their responses to abiotic stresses in < i > Gossypium hirsutum</i > L Plant Sci 2009 14 3 352 359 10.1016/j.plantsci.2008.12.003

[B15] Fujimoto SY Ohta M Usui A Shinshi H Ohme-Takagi M Arabidopsis ethylene-responsive element binding factors act as transcriptional activators or repressors of GCC box–mediated gene expression Plant Cell Online 2000 14 3 393 404 10.1105/tpc.12.3.393 PMC139839 10715325

[B16] Guo WJ David Ho TH An abscisic acid-induced protein, HVA22, inhibits gibberellin-mediated programmed cell death in cereal aleurone cells Plant Physiol 2008 14 4 1710 1722 10.1104/pp.108.120238 18583533 PMC2492636

[B17] Tp S Gibberellin-GID1-DELLA: a pivotal regulatory module for plant growth and development Plant Physiol 2010 14 2 567 570 10.1104/pp.110.161554 20921186 PMC2949019

[B18] Qu T Liu R Wang W An L Chen T Liu G Zhao Z Brassinosteroids regulate pectin methylesterase activity and < i > AtPME41</i > expression in < i > Arabidopsis</i > under chilling stress Cryobiology 2011 14 2 111 117 10.1016/j.cryobiol.2011.07.003 21819976

[B19] Van Aken O Giraud E Clifton R Whelan J Alternative oxidase: a target and regulator of stress responses Physiol Plant 2009 14 4 354 361 10.1111/j.1399-3054.2009.01240.x 19470093

[B20] Huang X Li J Bao F Zhang X Yang S A gain-of-function mutation in the arabidopsis disease resistance gene RPP4 confers sensitivity to low temperature Plant Physiol 2010 14 2 796 809 10.1104/pp.110.157610 20699401 PMC2949010

[B21] Dai X Cheng X Li Y Tang W Han L Differential expression of gibberellin 20 oxidase gene induced by abiotic stresses in Zoysiagrass (Zoysia japonica) Biologia 2012 14 4 681 688 10.2478/s11756-012-0048-3

[B22] Du Z Zhou X Ling Y Zhang Z Su Z agriGO: a GO analysis toolkit for the agricultural community Nucleic Acids Res 2010 14 Web Server W64 W70 10.1093/nar/gkq310 20435677 PMC2896167

[B23] Usadel B Poree F Nagel A Lohse M Czedik-Eysenberg A Stitt M A guide to using MapMan to visualize and compare Omics data in plants: a case study in the crop species, Maize Plant Cell Environ 2009 14 9 1211 1229 10.1111/j.1365-3040.2009.01978.x 19389052

[B24] Millar AH Whelan J Soole KL Day DA Organization and regulation of mitochondrial respiration in plants Annu Rev Plant Biol 2011 14 1 79 104 10.1146/annurev-arplant-042110-103857 21332361

[B25] Vanlerberghe GC McIntosh L Alternative oxidase: from gene to function Annu Rev Plant Biol 1997 14 1 703 734 10.1146/annurev.arplant.48.1.703 15012279

[B26] Arnholdt-Schmitt B Costa JH De Melo DF AOX – a functional marker for efficient cell reprogramming under stress? Trends Plant Sci 2006 14 6 281 287 10.1016/j.tplants.2006.05.001 16713324

[B27] Costa JH Mota EF Cambursano MV Lauxmann MA De Oliveira LMN Silva Lima MG Orellano EG Fernandes De Melo D Stress-induced co-expression of two alternative oxidase (VuAox1 and 2b) genes in Vigna unguiculata J Plant Physiol 2010 14 7 561 570 10.1016/j.jplph.2009.11.001 20005596

[B28] Fiorani F The alternative oxidase of plant mitochondria is involved in the acclimation of shoot growth at Low temperature. A study of arabidopsis AOX1a transgenic plants Plant Physiol 2005 14 4 1795 1805 10.1104/pp.105.070789 16299170 PMC1310560

[B29] Grellet Bournonville CF Díaz-Ricci JC Quantitative determination of superoxide in plant leaves using a modified NBT staining method Phytochem Anal 2011 14 3 268 271 10.1002/pca.1275 21360621

[B30] Gay C Collins J Gebicki JM Hydroperoxide assay with the ferric–xylenol orange complex Anal Biochem 1999 14 2 149 155 10.1006/abio.1999.4208 10469484

[B31] Park W Scheffler BE Bauer PJ Campbell BT Genome-wide identification of differentially expressed genes under water deficit stress in upland cotton (Gossypium hirsutum L.) BMC Plant Biol 2012 14 1 90 10.1186/1471-2229-12-90 22703539 PMC3438127

[B32] Yao D Zhang X Zhao X Liu C Wang C Zhang Z Zhang C Wei Q Wang Q Yan H Transcriptome analysis reveals salt-stress-regulated biological processes and key pathways in roots of cotton (Gossypium hirsutum L.) Genomics 2011 14 1 47 55 21569837 10.1016/j.ygeno.2011.04.007

[B33] Berg B Thanthiriwatte C Manda P Bridges SM Comparing gene annotation enrichment tools for functional modeling of agricultural microarray data BMC Bioinforma 2009 14 Suppl 11 S9 10.1186/1471-2105-10-S11-S9 PMC3226198 19811693

[B34] Yao D Zhang X Zhao X Liu C Wang C Zhang Z Zhang C Wei Q Wang Q Yan H Transcriptome analysis reveals salt-stress-regulated biological processes and key pathways in roots of cotton (Gossypium hirsutum L.) Genomics 2011 14 47 55 21569837 10.1016/j.ygeno.2011.04.007

[B35] Angert A Rodeghiero M Griffin K High alternative oxidase activity in cold soils and its implication to the Dole Effect Geophys Res Lett 2012 14 16 n/a-n/a

[B36] Armstrong AF Logan DC Tobin AK O’TOOLE P Atkin OK Heterogeneity of plant mitochondrial responses underpinning respiratory acclimation to the cold in Arabidopsis thaliana leaves Plant Cell Environ 2006 14 5 940 949 10.1111/j.1365-3040.2005.01475.x 17087477

[B37] Achard P Baghour M Chapple A Hedden P Van der Straeten D Genschik P Moritz T Harberd NP The plant stress hormone ethylene controls floral transition via DELLA-dependent regulation of floral meristem-identity genes Proc Natl Acad Sci 2007 14 15 6484 6489 10.1073/pnas.0610717104 17389366 PMC1851083

[B38] Cao W-H Liu J He X-J Mu R-L Zhou H-L Chen S-Y Zhang J-S Modulation of ethylene responses affects plant salt-stress responses Plant Physiol 2007 14 2 707 719 17189334 10.1104/pp.106.094292 PMC1803741

[B39] Wilkinson S Davies WJ Drought, ozone, ABA and ethylene: new insights from cell to plant to community Plant Cell Environ 2010 14 4 510 525 10.1111/j.1365-3040.2009.02052.x 19843256

[B40] Liu K Sun J Yao L Yuan Y Transcriptome analysis reveals critical genes and key pathways for early cotton fiber elongation in Ligon lintless-1 mutant Genomics 2012 14 1 42 50 10.1016/j.ygeno.2012.04.007 22576057

[B41] Meeuse BJ Thermogenic respiration in aroids Annu Rev Plant Physiol 1975 14 1 117 126 10.1146/annurev.pp.26.060175.001001

[B42] Cvetkovska M Vanlerberghe GC Alternative oxidase modulates leaf mitochondrial concentrations of superoxide and nitric oxide New Phytol 2012 14 1 32 39 10.1111/j.1469-8137.2012.04166.x 22537177

[B43] Maxwell DP Wang Y McIntosh L The alternative oxidase lowers mitochondrial reactive oxygen production in plant cells Proc Natl Acad Sci 1999 14 14 8271 8276 10.1073/pnas.96.14.8271 10393984 PMC22224

[B44] Rasmusson AG Fernie AR Van Dongen JT Alternative oxidase: a defence against metabolic fluctuations? Physiol Plant 2009 14 4 371 382 10.1111/j.1399-3054.2009.01252.x 19558416

[B45] Baena-González E Sheen J Convergent energy and stress signaling Trends Plant Sci 2008 14 9 474 482 10.1016/j.tplants.2008.06.006 18701338 PMC3075853

[B46] Harb A Krishnan A Ambavaram MMR Pereira A Molecular and physiological analysis of drought stress in arabidopsis reveals early responses leading to acclimation in plant growth Plant Physiol 2010 14 3 1254 1271 10.1104/pp.110.161752 20807999 PMC2971604

[B47] Provart NJ Gene expression phenotypes of arabidopsis associated with sensitivity to low temperatures Plant Physiol 2003 14 2 893 906 10.1104/pp.103.021261 12805619 PMC167029

[B48] Sagi M Plant respiratory burst oxidase homologs impinge on wound responsiveness and development in lycopersicon esculentum Plant Cell Online 2004 14 3 616 628 10.1105/tpc.019398 PMC385276 14973161

[B49] Seki M Narusaka M Ishida J Nanjo T Fujita M Oono Y Kamiya A Nakajima M Enju A Sakurai T Monitoring the expression profiles of 7000 Arabidopsis genes under drought, cold and high-salinity stresses using a full length cDNA microarray Plant J 2002 14 3 279 292 10.1046/j.1365-313X.2002.01359.x 12164808

[B50] Seo PJ Lee AK Xiang F Park CM Molecular and functional profiling of arabidopsis pathogenesis-related genes: insights into their roles in salt response of seed germination Plant Cell Physiol 2008 14 3 334 344 10.1093/pcp/pcn011 18203731

[B51] Caño-Delgado A Penfield S Smith C Catley M Bevan M Reduced cellulose synthesis invokes lignification and defense responses in Arabidopsis thaliana Plant J 2003 14 3 351 362 10.1046/j.1365-313X.2003.01729.x 12713541

[B52] Hernández-Blanco C Feng DX Hu J Sánchez-Vallet A Deslandes L Llorente F Berrocal-Lobo M Keller H Barlet X Sánchez-Rodríguez C Impairment of cellulose synthases required for Arabidopsis secondary cell wall formation enhances disease resistance Plant Cell Online 2007 14 3 890 903 10.1105/tpc.106.048058 PMC1867366 17351116

[B53] Hématy K Sado P-E Van Tuinen A Rochange S Desnos T Balzergue S Pelletier S Renou J-P Höfte H A receptor-like kinase mediates the response of < i > Arabidopsis</i > cells to the inhibition of cellulose synthesis Curr Biol 2007 14 11 922 931 10.1016/j.cub.2007.05.018 17540573

[B54] Schubert A Benedict C Berlin J Kohel R Cotton fiber development-kinetics of cell elongation and secondary wall thickening Crop Sci 1973 14 6 704 709 10.2135/cropsci1973.0011183X001300060035x

[B55] Xu B Huang Y Image analysis for cotton fibers part II: cross-sectional measurements Text Res J 2004 14 5 409 416 10.1177/004051750407400507

[B56] Brazma A Hingamp P Quackenbush J Sherlock G Spellman P Stoeckert C Aach J Ansorge W Ball CA Causton HC Minimum information about a microarray experiment (MIAME)—toward standards for microarray data Nat Genet 2001 14 4 365 371 10.1038/ng1201-365 11726920

[B57] Benedito VA Torres‒Jerez I Murray JD Andriankaja A Allen S Kakar K Wandrey M Verdier J Zuber H Ott T A gene expression atlas of the model legume Medicago truncatula Plant J 2008 14 3 504 513 10.1111/j.1365-313X.2008.03519.x 18410479

[B58] Conesa A Götz S García-Gómez JM Terol J Talón M Robles M Blast2GO: a universal tool for annotation, visualization and analysis in functional genomics research Bioinformatics 2005 14 18 3674 3676 10.1093/bioinformatics/bti610 16081474

[B59] Thimm O Bläsing O Gibon Y Nagel A Meyer S Krüger P Selbig J Müller LA Rhee SY Stitt M mapman: a user-driven tool to display genomics data sets onto diagrams of metabolic pathways and other biological processes Plant J 2004 14 6 914 939 10.1111/j.1365-313X.2004.02016.x 14996223

[B60] Paterson AH Wendel JF Gundlach H Guo H Jenkins J Jin D Llewellyn D Showmaker KC Shu S Udall J Repeated polyploidization of Gossypium genomes and the evolution of spinnable cotton fibres Nature 2012 14 7429 423 427 10.1038/nature11798 23257886

[B61] Bustin SA Benes V Garson JA Hellemans J Huggett J Kubista M Mueller R Nolan T Pfaffl MW Shipley GL The MIQE guidelines: minimum information for publication of quantitative real-time PCR experiments Clin Chem 2009 14 4 611 622 10.1373/clinchem.2008.112797 19246619

[B62] Daudi A Cheng Z O’Brien JA Mammarella N Khan S Ausubel FM Bolwell GP The apoplastic oxidative burst peroxidase in arabidopsis is a major component of pattern-triggered immunity Plant Cell 2012 14 1 275 287 10.1105/tpc.111.093039 22247251 PMC3289579

