# Supplementary material for: Functional analyses of cotton (Gossypium hirsutum L.) immature fiber (im) mutant infer that fiber cell wall development is associated with stress responses
Source: BMC Genomics. 2013 Dec 17;14:889. doi: 10.1186/1471-2164-14-889 (PMC3904472; doi:10.1186/1471-2164-14-889)
Supplement: Additional file 3 — Detailed annotation of the stimulus responding 103 DEGs s (Figure 5B) identified by PAGE analysis in the im mutant at 28 DPA. GO enrichment analysis of the stimulus responding 103 DEGs in the im mutant at 28 DPA and a magnified heatmap described at the Figure 5B. [file 1471-2164-14-889-S3.pdf]

103 DEGs identified as stimulus response genes by PAGE analysis in the im mutant fibers at 28 DPA

Probe ID	GO
**Ghi.9259.3.S1_x_at**	GO:0009536 GO:0042538 GO:0006982 GO:0005829 GO:0012505 GO:0008152 GO:0005886 GO:0046982
**Ghi.1478.1.S1_s_at**	GO:0005215 GO:0016021 GO:0006810 GO:0006950 GO:0005886
**GhiAffx.3411.1.A1_at**	GO:0042538 GO:0010200 GO:0009631 GO:0009611 GO:0009416 GO:0003676 GO:0009408 GO:0006979
**GhiAffx.8120.1.S1_at**	GO:0010043 GO:0006826
**GbaAffx.196.1.A1_s_at**	GO:0009735
**GbaAffx.201.1.S1_s_at**	GO:0006952 GO:0009607
**Ghi.9193.2.A1_at**	GO:0017053 GO:0009862 GO:0009864 GO:0050832 GO:0016564 GO:0005515 GO:0016481
**Gra.2050.1.S1_s_at**	GO:0050662 GO:0046369 GO:0009555 GO:0009225 GO:0005829 GO:0006950 GO:0009117 GO:0046983 GO:0003978
**GhiAffx.2436.2.A1_a_at**	GO:0003700 GO:0045449 GO:0005667 GO:0005515 GO:0009873
**GhiAffx.53494.1.S1_s_at**	GO:0009733 GO:0019827 GO:0051252 GO:0035198 GO:0005840 GO:0035197 GO:0035195 GO:0009616 GO:0009965 GO:0004521 GO:0009850 GO:0010218 GO:0048830 GO:0009793 GO:0009536 GO:0005515 GO:0005634 GO:0006446 GO:0003743
**Gra.3076.1.S1_x_at**	GO:0006570 GO:0042207 GO:0004334
**Gra.215.1.A1_at**	GO:0004365 GO:0051287 GO:0005829 GO:0009744 GO:0006979 GO:0006096 GO:0006094
**Ghi.10676.1.S1_s_at**	GO:0006952 GO:0009607
**GhiAffx.46685.1.S1_s_at**	GO:0006804 GO:0006979 GO:0004601
**Ghi.1665.1.S1_s_at**	GO:0005345 GO:0015211 GO:0006863 GO:0015860 GO:0016036 GO:0005887
**GraAffx.33059.2.S1_s_at**	GO:0005215 GO:0016021 GO:0006810 GO:0006950 GO:0005886
**GhiAffx.5920.3.A1_at**	GO:0006950 GO:0009536
**GhiAffx.30835.1.S1_s_at**	GO:0020037 GO:0006979 GO:0006804 GO:0005509 GO:0004601 GO:0006118
**Ghi.6953.1.S1_s_at**	GO:0031418 GO:0009835 GO:0009815 GO:0006950 GO:0009693 GO:0005506
**Ghi.1578.1.S1_s_at**	GO:0009570 GO:0009744 GO:0018316 GO:0019253 GO:0005515
**GhiAffx.8053.1.A1_at**	GO:0006952 GO:0009607
**Ghi.3315.1.A1_s_at**	GO:0010224
**Ghi.2804.1.S1_s_at**	GO:0009733 GO:0009718 GO:0008287 GO:0019497 GO:0009753 GO:0006771 GO:0003700 GO:0006470 GO:0006355 GO:0004722 GO:0019430 GO:0016036 GO:0009745 GO:0005667 GO:0006417 GO:0009651 GO:0046983 GO:0003993
**GhiAffx.24518.1.S1_s_at**	GO:0043234 GO:0009826 GO:0006468 GO:0010224 GO:0009742 GO:0009729 GO:0009647 GO:0004674 GO:0016023 GO:0001578 GO:0016021 GO:0010268 GO:0006952 GO:0005496 GO:0005886 GO:0004903 GO:0009069 GO:0046982 GO:0005768 GO:0005524 GO:0042803
**Ghi.8023.1.S1_at**	GO:0031418 GO:0009835 GO:0009815 GO:0006950 GO:0009693 GO:0005506
**Ghi.171.1.S1_at**	GO:0009733 GO:0009737 GO:0009735 GO:0009741 GO:0009739 GO:0009723 GO:0003677 GO:0009640
**Ghi.6551.1.S1_at**	GO:0005634 GO:0009416 GO:0007165 GO:0004871 GO:0005515
**GraAffx.14966.1.S1_s_at**	GO:0009733 GO:0019827 GO:0051252 GO:0035198 GO:0005840 GO:0035197 GO:0035195 GO:0009616 GO:0009965 GO:0004521 GO:0009850 GO:0010218 GO:0048830 GO:0009793 GO:0009536 GO:0005515 GO:0005634 GO:0006446 GO:0003743
**GhiAffx.42790.1.S1_at**	GO:0015893 GO:0016021 GO:0015717 GO:0009670 GO:0009507
**GhiAffx.41878.1.S1_at**	GO:0045087 GO:0004888 GO:0031224 GO:0007165
**GhiAffx.5920.1.S1_a_at**	GO:0006950 GO:0009536 GO:0005739
**Ghi.6462.1.S1_x_at**	GO:0006952 GO:0009607
**GhiAffx.10920.1.S1_s_at**	GO:0016021 GO:0015250 GO:0006833 GO:0006970 GO:0005886 GO:0009269
**GhiAffx.25343.1.S1_at**	GO:0020037 GO:0016023 GO:0006979 GO:0006804 GO:0004601 GO:0006118
**GhiAffx.53661.1.S1_s_at**	GO:0006656 GO:0040007 GO:0006499 GO:0019107 GO:0006950 GO:0042967 GO:0000234 GO:0005840
**Ghi.3235.1.A1_at**	GO:0035251 GO:0051555 GO:0051707
**Ghi.4957.1.S1_s_at**	GO:0006950

**GhiAffx.2754.1.A1_at**	GO:0005737 GO:0006950
**GhiAffx.62266.1.S1_at**	GO:0006355 GO:0005634 GO:0009734 GO:0046983 GO:0003677
**GhiAffx.24265.1.A1_at**	GO:0051607
**GhiAffx.44858.1.S1_at**	GO:0045261 GO:0015986 GO:0046933 GO:0005743 GO:0046961 GO:0046872 GO:0006979 GO:0005524
**Ghi.8485.2.A1_at**	GO:0009535 GO:0045017 GO:0009735 GO:0030912 GO:0009739 GO:0009416 GO:0009707 GO:0046509 GO:0009414 GO:0009409 GO:0009651
**GarAffx.19282.1.S1_s_at**	GO:0006952 GO:0009607
**Ghi.690.1.A1_s_at**	GO:0009739
**Ghi.8523.1.A1_at**	GO:0006979 GO:0005634 GO:0008270 GO:0008152 GO:0032440
**GhiAffx.26774.1.A1_s_at**	GO:0043153 GO:0005634 GO:0009637 GO:0045449 GO:0005515 GO:0010114 GO:0009908
**Ghi.9236.1.S1_at**	GO:0035251 GO:0051555 GO:0051707
**GhiAffx.41421.1.S1_at**	GO:0003700 GO:0009862 GO:0045449 GO:0005516 GO:0005667 GO:0042742
**GraAffx.4630.1.A1_s_at**	GO:0035251 GO:0051555 GO:0051707
**Ghi.3408.1.A1_at**	GO:0006810 GO:0016021 GO:0007585 GO:0045333 GO:0009409 GO:0005746 GO:0005506 GO:0006118 GO:0016491
**Ghi.7950.1.S1_at**	GO:0020037 GO:0006979 GO:0006804 GO:0005509 GO:0004601 GO:0006118
**GhiAffx.28714.1.S1_at**	GO:0005829 GO:0009735 GO:0006521 GO:0030139 GO:0007165 GO:0005886 GO:0019199 GO:0009986
**Ghi.1706.1.S1_s_at**	GO:0009753
**Ghi.10760.1.S1_s_at**	GO:0045430 GO:0005783 GO:0005634 GO:0009813 GO:0009705 GO:0010224
**GhiAffx.8267.1.A1_s_at**	GO:0042538 GO:0016020 GO:0009737 GO:0009414
**GhiAffx.58552.1.A1_s_at**	GO:0009536 GO:0009734 GO:0005634 GO:0006355 GO:0003677 GO:0046983 GO:0009908
**Ghi.3235.1.S1_s_at**	GO:0016758 GO:0051707 GO:0012505 GO:0008152 GO:0008194
**Ghi.8294.2.S1_at**	GO:0004103 GO:0006566 GO:0006563 GO:0006544 GO:0009611 GO:0046486
**Ghi.9261.2.A1_s_at**	GO:0009611 GO:0008152 GO:0005488
**Ghi.9259.1.S1_s_at**	GO:0009536 GO:0042538 GO:0006982 GO:0005829 GO:0003824 GO:0012505 GO:0005886 GO:0008615 GO:0046982
**Ghi.10646.1.S1_s_at**	GO:0009816
**Gra.2508.1.A1_x_at**	GO:0009629
**Ghi.440.1.S1_s_at**	GO:0019852 GO:0006631 GO:0006574 GO:0004029 GO:0006560 GO:0006552 GO:0006547 GO:0046251 GO:0019482 GO:0006568 GO:0019260 GO:0006554 GO:0006550 GO:0006525 GO:0006699 GO:0046486 GO:0006096 GO:0006094
**Ghi.6484.1.S1_at**	GO:0009733
**GhiAffx.5920.2.S1_x_at**	GO:0006950 GO:0005739
**Ghi.9264.2.A1_s_at**	GO:0006810 GO:0016021 GO:0007585 GO:0009916 GO:0009409 GO:0005746 GO:0010230 GO:0005506 GO:0006118
**GraAffx.20510.2.A1_s_at**	GO:0042493 GO:0006952 GO:0009411 GO:0012505 GO:0005515 GO:0007165
**Ghi.954.1.A1_s_at**	GO:0048316 GO:0006560 GO:0008792 GO:0006527 GO:0009651 GO:0008295
**GraAffx.11285.1.A1_s_at**	GO:0016021 GO:0015250 GO:0006833 GO:0006970 GO:0005886 GO:0009269
**Ghi.6559.1.S1_at**	GO:0020037 GO:0016023 GO:0004601 GO:0006804 GO:0005509 GO:0006979 GO:0006118 GO:0012505
**Ghi.7872.1.S1_s_at**	GO:0009753
**GbaAffx.207.1.S1_s_at**	GO:0006952 GO:0009607
**Ghi.7615.1.S1_s_at**	GO:0009638 GO:0009734 GO:0006355 GO:0005634 GO:0003677 GO:0009723 GO:0048366 GO:0010386 GO:0016563 GO:0009785 GO:0046983 GO:0009630 GO:0045941
**Gra.2513.1.A1_s_at**	GO:0005737 GO:0006457 GO:0051082 GO:0006950 GO:0012505 GO:0005524
**GhiAffx.9838.1.S1_at**	GO:0009651
**Ghi.8364.1.A1_at**	GO:0009409
**GhiAffx.10836.1.A1_at**	GO:0005737 GO:0010224 GO:0050645 GO:0050284 GO:0008152
**GhiAffx.41577.1.S1_s_at**	GO:0009408
**GhiAffx.52815.1.S1_at**	GO:0015250 GO:0016021 GO:0006833 GO:0009414 GO:0005886

**GhiAffx.18100.1.S1_at**	GO:0003723 GO:0009704 GO:0006412
**GhiAffx.52609.1.S1_at**	GO:0015293 GO:0009734 GO:0016021 GO:0006865 GO:0009624 GO:0005886
**Ghi.6485.1.S1_s_at**	GO:0006952 GO:0009607
**GhiAffx.17865.1.S1_at**	GO:0006457 GO:0005739 GO:0006950 GO:0051082 GO:0046685 GO:0005524
**Ghi.10779.1.S1_s_at**	GO:0004365 GO:0047100 GO:0051287 GO:0009744 GO:0009416 GO:0005515 GO:0009570 GO:0006096 GO:0019253 GO:0006094
**Ghi.1970.1.S1_s_at**	GO:0006950
**Ghi.7497.1.S1_s_at**	GO:0009651
**Gra.202.3.S1_s_at**	GO:0009395 GO:0016020 GO:0012501 GO:0005509 GO:0009409 GO:0004630 GO:0046470 GO:0046473
**Ghi.3555.1.A1_at**	GO:0009834 GO:0009863 GO:0005982 GO:0005886 GO:0009867 GO:0005985 GO:0030244 GO:0050832 GO:0006011 GO:0016760 GO:0042742 GO:0009414 GO:0009873 GO:0006970 GO:0010116
**GhiAffx.24133.1.S1_at**	GO:0006952 GO:0009807 GO:0012505
**GhiAffx.6043.1.A1_at**	GO:0020037 GO:0016165 GO:0006804 GO:0004601 GO:0008219 GO:0051707 GO:0006979 GO:0006118
**GhiAffx.36025.1.S1_at**	GO:0016023 GO:0009536 GO:0019497 GO:0003993 GO:0006771
**Ghi.6523.1.S1_s_at**	GO:0006952 GO:0009607
**Gra.302.1.S1_s_at**	GO:0009735
**Ghi.4137.1.S1_at**	GO:0006952 GO:0050660 GO:0016021 GO:0016174 GO:0004601 GO:0006804 GO:0009060 GO:0006800 GO:0005509 GO:0009408 GO:0005506 GO:0043069 GO:0006979 GO:0006118
**Ghi.8273.1.S1_s_at**	GO:0009733 GO:0016481 GO:0003960 GO:0006118 GO:0010181
**Ghi.7874.1.S1_s_at**	GO:0009735
**GhiAffx.7280.1.S1_at**	GO:0004497 GO:0020037 GO:0016132 GO:0016023 GO:0010268 GO:0010224 GO:0006118
**GraAffx.27655.1.S1_s_at**	GO:0016023 GO:0006810 GO:0005938 GO:0005887 GO:0009734
**GhiAffx.25159.1.A1_at**	GO:0009733
**GraAffx.30479.1.A1_a_at**	GO:0050896
**GhiAffx.25696.1.S1_at**	GO:0006950
**GarAffx.19282.1.S1_x_at**	GO:0006952 GO:0009607

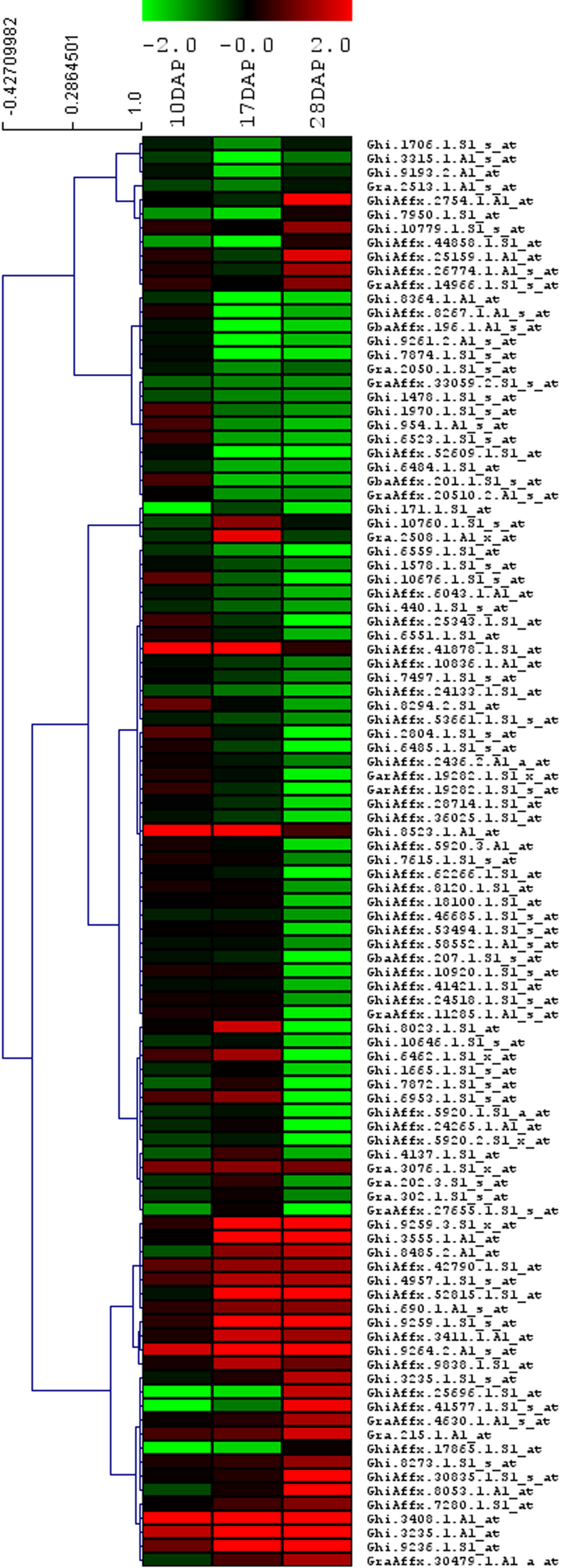
-0.42709982
0.2864501
1.0
-2.0
-0.0
2.0
10DAP
17DAP
28DAP
Ghi.1706.1.S1_s_at
Ghi.3315.1.A1_s_at
Ghi.9193.2.A1_at
Gra.2513.1.A1_s_at
GhiAffx.2754.1.A1_at
Ghi.7950.1.S1_at
Ghi.10779.1.S1_s_at
GhiAffx.44858.1.S1_at
GhiAffx.25159.1.A1_at
GhiAffx.26774.1.A1_s_at
GraAffx.14966.1.S1_s_at
Ghi.8364.1.A1_at
GhiAffx.8267.1.A1_s_at
GbaAffx.196.1.A1_s_at
Ghi.9261.2.A1_s_at
Ghi.7874.1.S1_s_at
Gra.2050.1.S1_s_at
GraAffx.33059.2.S1_s_at
Ghi.1478.1.S1_s_at
Ghi.1970.1.S1_s_at
Ghi.954.1.A1_s_at
Ghi.6523.1.S1_s_at
GhiAffx.52609.1.S1_at
Ghi.6484.1.S1_at
GbaAffx.201.1.S1_s_at
GraAffx.20510.2.A1_s_at
Ghi.171.1.S1_at
Ghi.10760.1.S1_s_at
Gra.2508.1.A1_x_at
Ghi.6559.1.S1_at
Ghi.1578.1.S1_s_at
Ghi.10676.1.S1_s_at
GhiAffx.6043.1.A1_at
Ghi.440.1.S1_s_at
GhiAffx.25343.1.S1_at
Ghi.6551.1.S1_at
GhiAffx.41878.1.S1_at
GhiAffx.10836.1.A1_at
Ghi.7497.1.S1_s_at
GhiAffx.24133.1.S1_at
Ghi.8294.2.S1_at
GhiAffx.53661.1.S1_s_at
Ghi.2804.1.S1_s_at
Ghi.6485.1.S1_s_at
GhiAffx.2436.2.A1_a_at
GarAffx.19282.1.S1_x_at
GarAffx.19282.1.S1_s_at
GhiAffx.28714.1.S1_at
GhiAffx.36025.1.S1_at
Ghi.8523.1.A1_at
GhiAffx.5920.3.A1_at
Ghi.7615.1.S1_s_at
GhiAffx.62266.1.S1_at
GhiAffx.8120.1.S1_at
GhiAffx.18100.1.S1_at
GhiAffx.46685.1.S1_s_at
GhiAffx.53494.1.S1_s_at
GhiAffx.58552.1.A1_s_at
GbaAffx.207.1.S1_s_at
GhiAffx.10920.1.S1_s_at
GhiAffx.41421.1.S1_at
GhiAffx.24518.1.S1_s_at
GraAffx.11285.1.A1_s_at
Ghi.8023.1.S1_at
Ghi.10646.1.S1_s_at
Ghi.6462.1.S1_x_at
Ghi.1665.1.S1_s_at
Ghi.7872.1.S1_s_at
Ghi.6953.1.S1_s_at
GhiAffx.5920.1.S1_a_at
GhiAffx.24265.1.A1_at
GhiAffx.5920.2.S1_x_at
Ghi.4137.1.S1_at
Gra.3076.1.S1_x_at
Gra.202.3.S1_s_at
Gra.302.1.S1_s_at
GraAffx.27655.1.S1_s_at
Ghi.9259.3.S1_x_at
Ghi.3555.1.A1_at
Ghi.8485.2.A1_at
GhiAffx.42790.1.S1_at
Ghi.4957.1.S1_s_at
GhiAffx.52815.1.S1_at
Ghi.690.1.A1_s_at
Ghi.9259.1.S1_s_at
GhiAffx.3411.1.A1_at
Ghi.9264.2.A1_s_at
GhiAffx.9838.1.S1_at
Ghi.3235.1.S1_s_at
GhiAffx.25696.1.S1_at
GhiAffx.41577.1.S1_s_at
GraAffx.4630.1.A1_s_at
Gra.215.1.A1_at
GhiAffx.17865.1.S1_at
Ghi.8273.1.S1_s_at
GhiAffx.30835.1.S1_s_at
GhiAffx.8053.1.A1_at
GhiAffx.7280.1.S1_at
Ghi.3408.1.A1_at
Ghi.3235.1.A1_at
Ghi.9236.1.S1_at
GraAffx.30479.1.A1_a_at
