# Supplementary material for: Functional analyses of cotton (Gossypium hirsutum L.) immature fiber (im) mutant infer that fiber cell wall development is associated with stress responses
Source: BMC Genomics. 2013 Dec 17;14:889. doi: 10.1186/1471-2164-14-889 (PMC3904472; doi:10.1186/1471-2164-14-889)
Supplement: Additional file 7 — qPCR primer sequences. Forward and reverse primer sequences for quantitative PCR analysis. [file 1471-2164-14-889-S7.docx]

|  | **Names** | **Forward primers ( 5′-3′)** | **Reverse primers ( 5′-3′)** |
| --- | --- | --- | --- |
| **Down-regulated genes in *im* mutant** | *NAC 11-like* | CAAGGAGTGGTCATGGTGGTAA | AGTTGCCTTCCAATACCCTCTCT |
|  | *NAC 2* | GGCGCCATCGAGAAACAG | CGGCAGAGCTTTTGCCAAT |
|  | *ERF3* | TGAAGCCGCCAAGCGTAT | GGGCGTTTGAGGGAAGTTG |
|  | *GA receptor* | GCCGATCGCCTGAACATAGA | AGCCGCCCAACCATCA |
|  | *HVA22* | CCCCTATATGCCTCGGTGATAG | AGCAAGCCACTGTTCATCATCTT |
|  | *Pectin methylesterase* | CCCTCCCGGCAGTACCA | CGTTGTATTTTGCCGAGTATGC |
| **Up-regulated**  **genes in *im* mutant** | *Csl E* | CATTTGCATATGTTGCCTTTGTACA | TGCCTCCGCACCATAGAAA |
|  | *TIR-NBS-LRR* | AAGCTTCCAAGAGGAGATGAAATTT | ACCCTCGCTCCTTCAATGG |
|  | *GA 20 oxidase* | GCCCTTTCGTGGCTATTTCA | CTTCCATCGCCAAGCTTTG |
|  | *Alternative oxidase* | GGCGCTTGCCCCCTAA | TCTGCCCTCACAGCCAAAAC |
|  | *Allyl alcohol dehydrogenase* | GCCGATTACTATCCACAGTATTCGA | CCACATACTTTATCTTCCCTTCTCTAATG |
|  | *α-Expansin 8* | GGATGGCTACCTATGTCCAGAAA | GCTGGCAGTCACTTTAGAAAGAGA |
| **Reference genes** | *18S rRNA* | CGTCCCTGCCCTTTGTACA | AACACTTCACCGGACCATTCA |
|  | *ubiquitin-conjugating protein* | CGGAAAGAGGTGAAGATGTCAAC | GGATCTTGCTGCAACCTCTTAAA |
|  | *α-tubulin 4* | GATCTCGCTGCCCTGGAA | ACCAGACTCAGCGCCAACTT |
